# Zebrafish dspb-/- mutant as a model for non-dilated left ventricular cardiomyopathy: exploring cardiac dysfunction and exercise modulation

**DOI:** 10.1186/s43556-026-00476-7

**Published:** 2026-06-15

**Authors:** Serena Munteanu, Jesús Wagih Gómez, Ángel Júdez Serrano, Daniel Saura Espín, Juan José Santos Mateo, María Elisa Nicolás Rocamora, Cristina Gil Ortuño, Ángel Bernabé García, Juan Ramon Gimeno-Blanes, María Luisa Cayuela Fuentes, María Sabater Molina

**Affiliations:** 1 Cardiogenetic Laboratory, Biomedical Research Institute of Murcia - Pascual Parrilla (IMIB-PP), University of Murcia, Murcia, Spain; 2 Inherited Cardiac Disease Unit (CSUR), Virgen de la Arrixaca University Hospital, Murcia, Spain; 3 European Reference Network for Rare and Low Prevalence Complex Diseases of the Heart (ERN-Guard Heart), Amsterdam, The Netherlands; 4 Department of Internal Medicine (Cardiology), University of Murcia, Murcia, Spain; 5 Cardiogenetic Laboratory, Murcia Biomedical Research Institute Pascual Parrilla (IMIB-PP), Murcia, Spain; 6 Murcia Biomedical Research Institute Pascual Parrilla (IMIB-PP), Murcia, Spain; 7 General Surgery Department, Virgen de La Arrixaca University Hospital, Murcia, Spain; 8 Department of Legal and Forensic Medicine, Faculty of Medicine, University of Murcia, Murcia, Spain

**Keywords:** Desmoplakin, Truncating variants, Physical activity, Cardiac function, Non-dilated cardiomyopathy, Zebrafish model

## Abstract

Truncating variants in the DSP gene are associated with non-dilated left ventricular cardiomyopathy (ND-LVC), characterized by impaired ventricular function without dilation. The influence of physical activity in disease onset, severity, and progression in DSP variant carriers remains unclear. This study aimed to generate a zebrafish model carrying a human DSP truncating variant to characterize homozygous dspb–/– mutants in terms of cardiac function, structure, and gene expression. The impact of moderate and endurance exercise was additionally evaluated. Clinical data from human carriers were also analysed to assess the relationship between physical activity, age at diagnosis, disease severity and arrhythmic events. A CRISPR/Cas9-generated dspb–/– zebrafish model (p.T449fs*) was used to characterize structural, functional, and molecular phenotypes. Moderate exercise training protocols were applied to assess their effect on cardiac function, endurance capacity, and survival. Physically active human carriers were diagnosed at a younger age but did not show increased disease severity or events rates. Zebrafish mutants exhibited baseline systolic dysfunction, sarcomeric disorganization, and signaling pathway dysregulation. Importantly, moderate exercise partially restored cardiac function and improved endurance without increasing mortality. These findings indicate that exercise may serve as a phenotypic modifier in DSP-related ND-LVC, enhancing functional outcomes without accelerating disease progression. The dspb–/– zebrafish model provides a robust translational platform to investigate the interactions between lifestyle factors and the pathogenesis of inherited desmosomal cardiomyopathies.

**Supplementary Information:**

The online version contains supplementary material available at 10.1186/s43556-026-00476-7.

## Introduction

ND-LVC represents a recently proposed phenotypic classification of cardiomyopathies, characterized by functional or structural impairment of the left ventricle in the absence of ventricular dilatation. This concept reflects an evolution in the understanding of hereditary cardiomyopathies, emphasizing the importance of mechanisms that disrupt diastolic or systolic function in the absence of classical dilated phenotypes. ND-LVC is frequently associated with pathogenic variants in genes involved in cytoskeletal structure and intercalated disc integrity, such as desmoplakin (*DSP)*, filamin-C (*FLNC*—truncating variants), desmin (*DES)*, lamin A/C (*LMNA)*, and phospholamban (*PLN)* [1]. Disease-causing variants in these genes have been linked to cardiomyopathies with an increased risk of arrhythmias and sudden cardiac death (SCD), particularly in young individuals and athletes. DSP variants, in particular, are associated with a distinctive phenotype characterized by a high prevalence of LV fibrosis and myocarditis [1, 2]. Although physical exercise is generally considered beneficial, its role in cardiomyopathy patients remains controversial, as intense or prolonged exercise may promote disease progression and can trigger life-threatening arrhythmias [3].

Desmosomes are composed of transmembrane cadherins, armadillo proteins, and the plakin protein desmoplakin, which plays a central role in maintaining both intercellular adhesion and cytoskeletal integrity in cardiac muscle. This is due to the close association of desmosomes with intermediate filaments [4]. Desmoplakin was the first desmosomal gene to be linked to autosomal dominant arrhythmogenic cardiomyopathy (ACM) [5]. A heterozygous DSP nonsense variant (NM_004415.4: c.1339C > T, p.Q447*) with a founder effect has been identified in the Region of Murcia, Spain, with 39 carriers from 5 families reported to date. This variant shows high penetrance (83%) by the age of 40 years old. The affected residue is located within one of the globular head domains of desmoplakin, which mediates interactions with catenin proteins [1, 6].

The zebrafish model has been widely used to understand the biological function of genes orthologous to those implicated in human disease [7, 8]. Genes involved in cell–cell adhesion, which are associated with ACM and ND-LVC when altered, have also been studied in zebrafish [9]. Orthologues for key desmosomal genes are present in zebrafish, including desmocollin (*zfDsc*), which shares 68% amino acid identity, and two desmoglein genes (*zfDsgα* and *zfDsgβ*), which share approximately 61% identity with their human counterparts. Regarding desmoplakin, zebrafish possess two paralogues, dspa and dspb, which together represent the functional equivalent of human DSP. These genes share approximately 49% and 44% identity with human DSP, respectively, and exhibit significant functional conservation, supporting their use in modeling DSP-related cardiomyopathies [10].

In this study, we aimed to reproduce a human *DSP* truncating variant in zebrafish to generate an ND-LVC model. We targeted exon 10 of the zebrafish *dspb* gene to induce a mutation resulting in a premature stop codon analogous to the human variant. After generating the *dspb*^*−/−*^ p.T449fs* (c.1340_1352delTGGCCACTTACAG) model using CRISPR/Cas9, our primary objective was to phenotypically characterize the homozygous mutant zebrafish focusing on cardiac function and histological features. Additionally, we assessed viability, gene expression, and evaluated the impact of both moderate and endurance exercise.

## Results

### Clinical profile and impact of physical activity in DSP truncating variant carriers

**Fig. 1:**
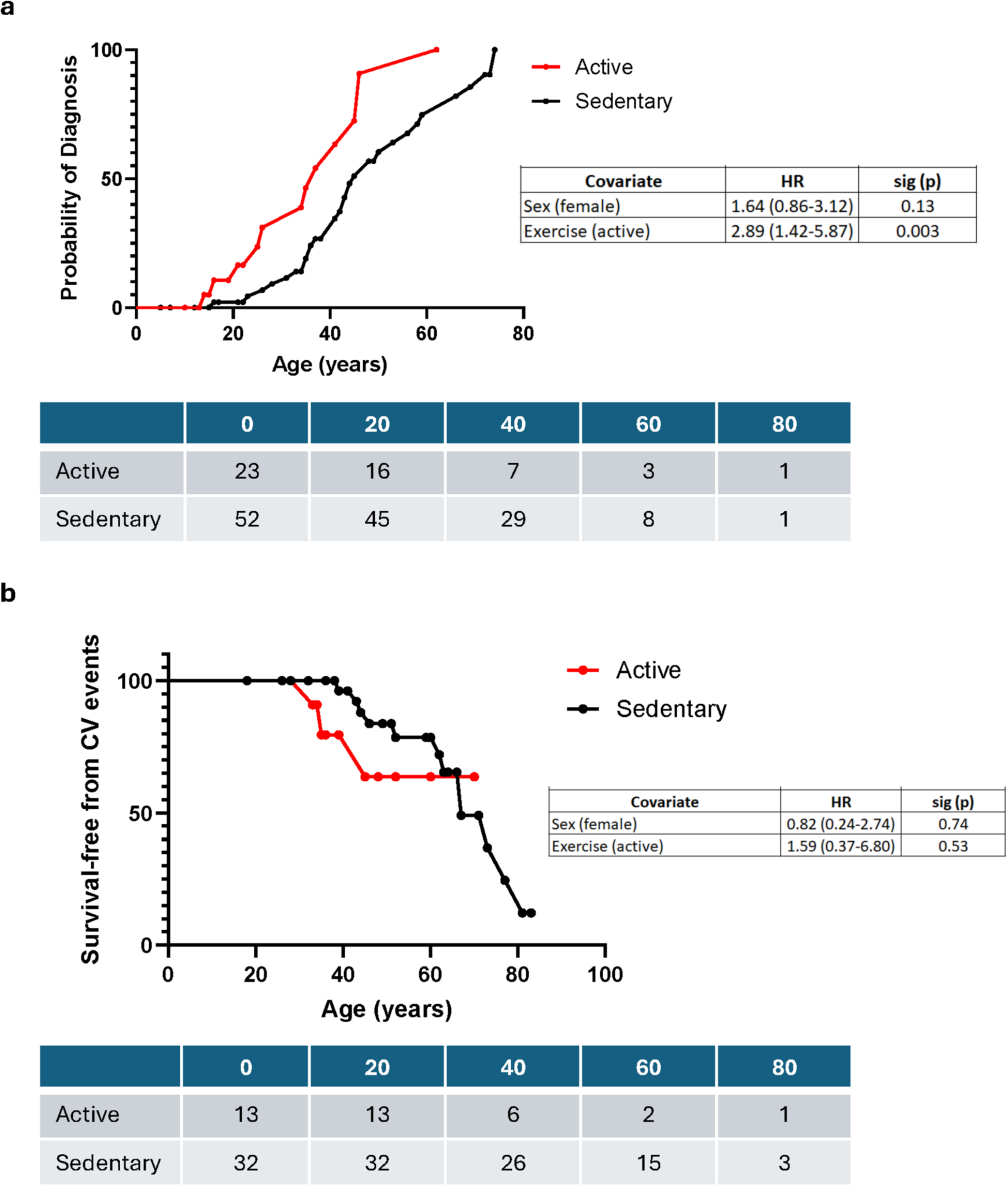
Age-dependent penetrance and event free survival in DSP truncating variant carriers according to physical activity. Kaplan–Meier curves show earlier age at diagnosis in individuals with moderate/intense physical activity compared with sedentary/light individuals **a**, while no differences were observed in event-free survival **b** Adjusted hazard ratios were obtained using multivariable Cox regression. Numbers at risk are shown below

Among the 45 affected carriers of *DSP* truncating variants, 13 (28.9%) reported engaging in moderate or intense physical activity, while 32 (71.1%) were classified as sedentary. Individuals in the active group were significantly younger at diagnosis (33.0 ± 13.5 vs. 47.1 ± 13.7 years, *p* = 0.008) and were more frequently male (76.9% vs. 25.5%, *p* = 0.002) (Table S1). Sedentary patients more often presented with symptoms (50.0% vs. 7.6%, *p* = 0.012), particularly palpitations (40.6% vs. 0.0%, *p* = 0.008). A total of 16 individuals (35.5%) met diagnostic criteria for ND-LVC (11 [68.7%] *vs* 5 [31.2%] in the sedentary and moderate/intense groups, respectively). Cardiac imaging parameters, including LVEF, ventricular dimensions, and LGE on CMR, did not differ significantly between groups. Similarly, no differences were observed in the rates of arrhythmias, ICD implantation, or major cardiac events. Kaplan–Meier analysis confirmed a significantly earlier age at diagnosis in carriers with a history of moderate/intense exercise compared to sedentary individuals (log-rank *p* = 0.006; HR 2.89 [95% CI: 1.42–5.87 ]; Fig. 1a). In contrast, no significant differences in event-free survival were observed between active and sedentary groups (Fig. 1b), with no significant association between physical activity and major adverse outcomes.

### Generation and validation of a dspb-/- zebrafish model mimicking the human ND-LVC variant

CRISPR/Cas9 genome editing was used to introduce a targeted variant in exon 10 of the zebrafish *dspb* gene. The resulting allele carries a 13 bp deletion (c.1340_1352delTGGCCACTTACAG), as confirmed by Sanger sequencing. This deletion causes a frameshift at codon 449, generating a premature stop codon (p.T449fs*), homologous to the human *DSP* nonsense variant p.Q447* associated with ND-LVC (Fig. S1).

### Developmental impairment and reduced viability in dspb-/- zebrafish

**Fig. 2:**
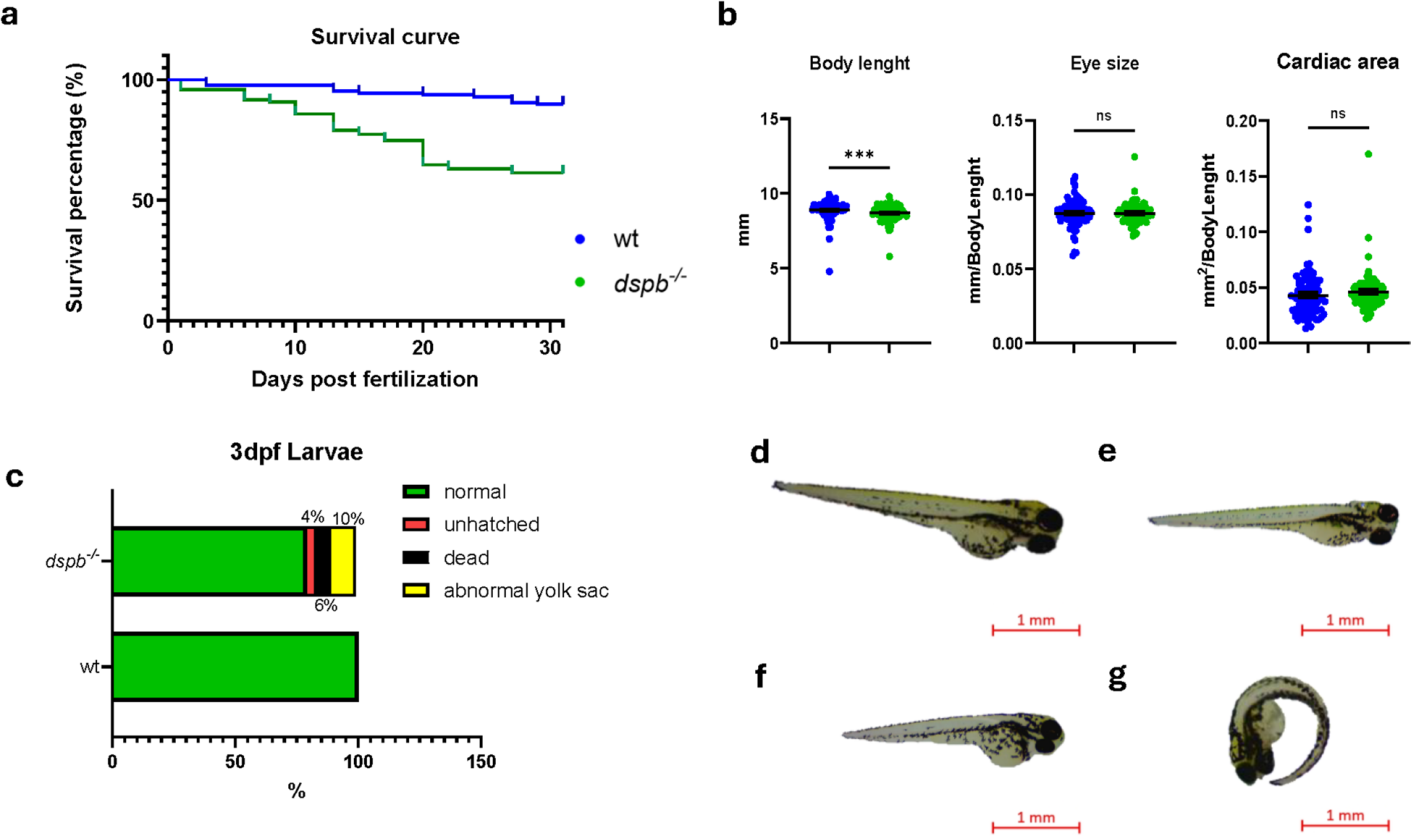
Impaired survival and development in *dspb*^*−/−*^ larvae. **a** Survival analysis of *dspb*^-/-^ vs. wt group (HR 4.1 [95% CI: 2.4-6.9]; **** *p* < 0.0001). Test: Simple Survival Analysis (Kaplan-Meier) with Log-rank (Mantel-Cox test) and Hazard Ratio (Mantel-Haenszel). Comparison of larvae at 3 dpf. **b** Body measurements showed significant differences in body lengths between wt and *dspb*^-/-^ group (* *p* < 0.05), with *dspb*^-/-^ larvae being generally shorter than the control group. However, eye size and cardiac area data, normalized with each embryo’s body length, showed no statistically significant differences. While wt group showed 100% normal embryos (**c**, **d**), *dspb*^-/-^ group consisted of 80% normal embryos (**c**, **e**), 10% embryos showing an abnormal yolk sac (**c**, **f**), 4% unhatched eggs (**c**, **g**), and 6% dead larvae (*** *p* < 0.0002, ns – not significant) Sample size: *n*=100. Test: Student’s t-test

To contextualize the phenotypes observed in the homozygous line, we first evaluated the consequences of partial *dspb* loss. Heterozygous *dspb*^+*/−*^ embryos and larvae showed no significant differences in survival, heart rate, or gross morphology compared with wt siblings (Fig. S2), indicating that a single functional allele preserves early developmental and cardiac homeostasis. In contrast, homozygous *dspb*^−/−^ mutants exhibited markedly reduced viability, with premature lethality and significantly lower survival rates compared with controls (Fig. 2a). These findings indicate that full *dspb* function is essential for normal embryonic development and early cardiac stability. At 3 dpf, *dspb*^*−/−*^ embryos showed a significantly shorter body length compared with controls (wt 8.89 ± 0.06 vs. *dspb*^*−/−*^8.68 ± 0.05,* p* < 0.00002, *n* = 100). However, no differences were observed in eye size or cardiac area after normalization to body length (Fig. 2b). While 100% of wt embryos were morphologically normal, the *dspb*^*−/−*^ group showed 80% normal larvae, 6% dead larvae, 4% unhatched eggs and 10% larvae with enlarged yolk sacs (Fig. 2c-g).

### Dysregulation of desmosomal genes and key signaling pathways across development

In zebrafish larvae at 20 dpf, *dspb*^*−/−*^ mutants exhibited lower expression of *dspb* compared with wt larvae. Expression of *pkp2* was also reduced, whereas *dspa* expression remained unchanged (Fig. S3a). Gene expression analysis across adult tissues revealed tissue-specific alterations (Fig. S3b-d). In cardiac tissue, *dspb* expression was reduced, whereas *pkp2* and *dspa* expression was increased, suggesting compensatory mechanisms. Similar patterns were observed in skin tissue. In tail tissue, *dspb* expression was also reduced; however, *pkp2* expression was not increased, while *dspa* expression was significantly elevated.

**Fig. 3:**
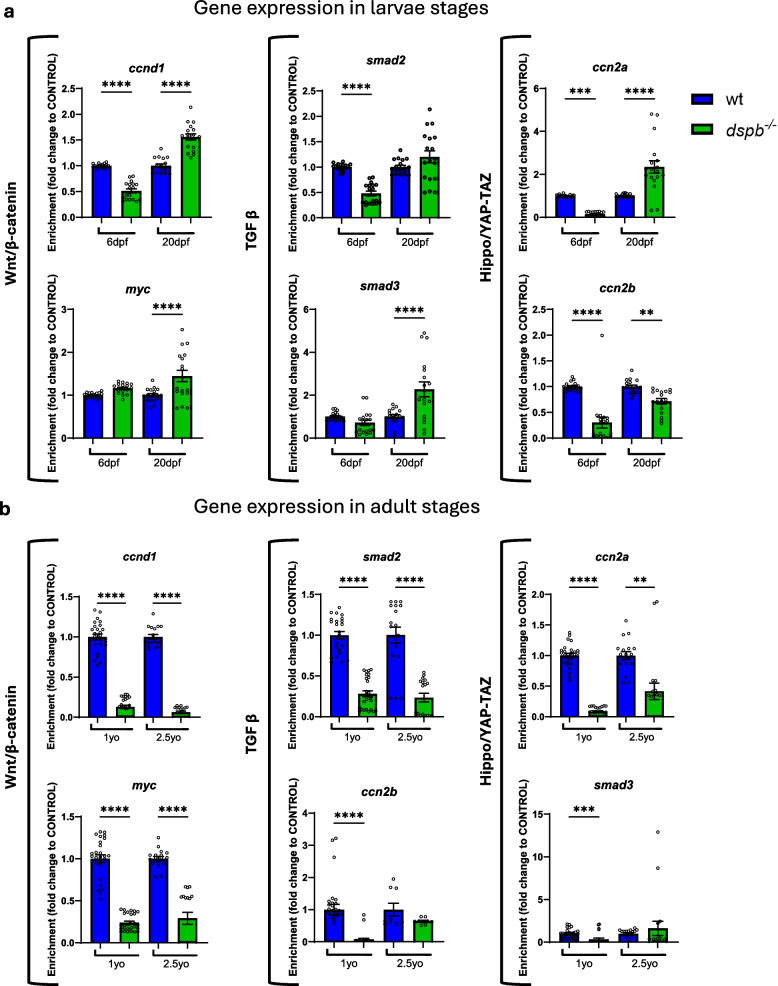
Gene expression analysis in wt and *dspb*^*−/−*^. **a** larvae and **b** adults at different stages, to assess the expression levels of key genes in fundamental signaling pathways. To determine the enrichment of each gene in each age group, constitutive gene rps11 was used as control to calculate the fold change (**p < 0.01, ***p < 0.001, ****p < 0.0001) Error bars: SEM. Number of experiments: 2 replicates for larvae stages; 3 replicates for 1 year-old; 2 replicates for 2.5-year-old. Test: Two-way ANOVA. Sample size: Pool of 20 larvae for 6 dpf, 10 larvae for 20 dpf, pool of 3 hearts for each adult group

qPCR analysis was performed to assess the expression of genes involved in Wnt/β-catenin (*ccnd1, myc*), TGF-β (*smad2, smad3*), and Hippo/YAP-TAZ (*ccn2a, ccn2b*) signaling pathways in both wt and *dspb*^*−/−*^ zebrafish hearts at different developmental stages. At 6 dpf, *ccnd1* was significantly downregulated in *dspb*^*−/−*^ larvae (*p* < 0.0001), whereas *myc* expression remained unchanged. Both genes were upregulated at 20 dpf (*p* < 0.0001). In the TGF-β pathway, *smad2* was downregulated at 6 dpf (*p* < 0.0001), and returned to baseline levels at 20 dpf, while *smad3* showed no change at 6 dpf but was upregulated at 20 dpf (*p* < 0.0001). Hippo/YAP-TAZ signaling exhibited dynamic regulation: *ccn2a* was downregulated at 6 dpf (*p* < 0.001) and upregulated at 20 dpf (*p* < 0.0001), whereas *ccn2b* remained consistently downregulated, with partial recovery at 20 dpf (Fig. 3a; Table S2). In adult hearts (1-year-old and 2.5-year-old), *ccnd1* and *myc* were significantly reduced in *dspb*^*−/−*^ mutants across both age groups. Similarly, *smad2* and *smad3* were markedly downregulated, with partial recovery of *smad3* in 2.5-year-old mutants. In the Hippo/YAP-TAZ pathway, *ccn2a* and *ccn2b* were downregulated at 1 year, with *ccn2a* remaining significantly reduced and *ccn2b* partially recovering at 2.5 years (Fig. 3b and Table S2).

### Early-onset and progressive cardiac dysfunction in dspb-/- zebrafish

**Fig. 4:**
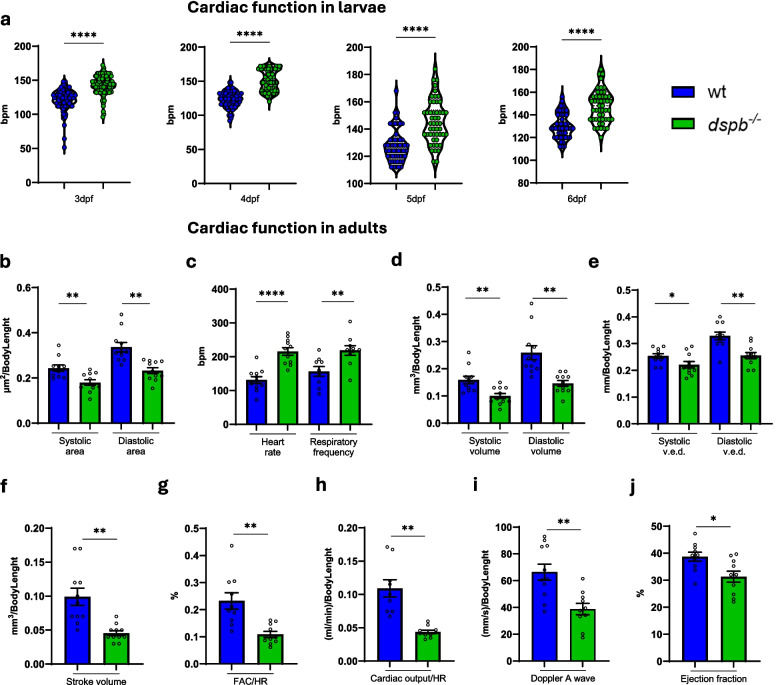
Cardiac function in larvae and adults. **a** Heart rate in dsp mutant larvae between 3–6 dpf, measured by daily counting heart rates of larvae and comparing *dspb*^*−/−*^ mean to the wt mean (****p < 0.0001) (bpm: Beats per minute) Sample size: 3 dpf *n* = 100; 4 dpf *n* = 70; 5 dpf *n* = 50; 6 dpf = 50 Error bars: SEM. Test: Student’s t-test**. b-k** Cardiac function in wt and *dspb*^*−/−*^ adults, pre-training, measured by echocardiography. Significant differences were seen between the two groups in (**b**) higher heart rate and respiratory frequency for *dspb*^*−/−*^ mutants, **c** smaller systolic and diastolic areas for *dspb*^*−/−*^*,***d** smaller systolic and diastolic volumes for *dspb*^*−/−*^, **e** smaller systolic and diastolic ventricular entry for *dspb*^*−/−*^, **f** reduced stroke volume, **g** reduced cardiac output, **h** reduced ejection fraction for *dspb*^*−/−*^, **i** reduced fractional area change for *dspb*^*−/−*^ and **k** lower values for atrial contraction in *dspb*^*−/−*^ (*p < 0.05, **p < 0.01, ****p < 0.0001) Sample size: *n* = 11. Error bars: SEM. Test: Unpaired Multiple T-test with Bonferroni correction

At 3 dpf, *dspb*^*−/−*^ larvae exhibited a significantly higher heart rate compared with wt controls (wt 122.10 ± 1.53 *vs dspb*^*−/−*^143.20 ± 1.34 bpm, *p* < 0.0001). This tachycardic phenotype persisted from 3 to 6 dpf (Fig. 4a). In adult zebrafish (8 months old), echocardiographic analysis revealed significant differences between groups (Fig. 4b-k; Table S3). The *dspb*^*−/−*^ group showed higher heart rate and respiratory frequency compared with wt controls. Additionally, systolic and diastolic areas were significantly reduced in mutants. Both systolic and diastolic volumes were also decreased, along with ventricular inflow parameters. The reduced stroke volume in mutants was associated with decreased cardiac output, ejection fraction, and fractional area change. Doppler A wave values were also significantly lower in *dspb*^*−/−*^ fish. Overall, these findings suggest that the *dspb*^*−/−*^ variant leads to impaired cardiac function and altered physiological parameters in adult zebrafish.

### Structural myocardial abnormalities revealed by histology and ultrastructure

**Fig. 5:**
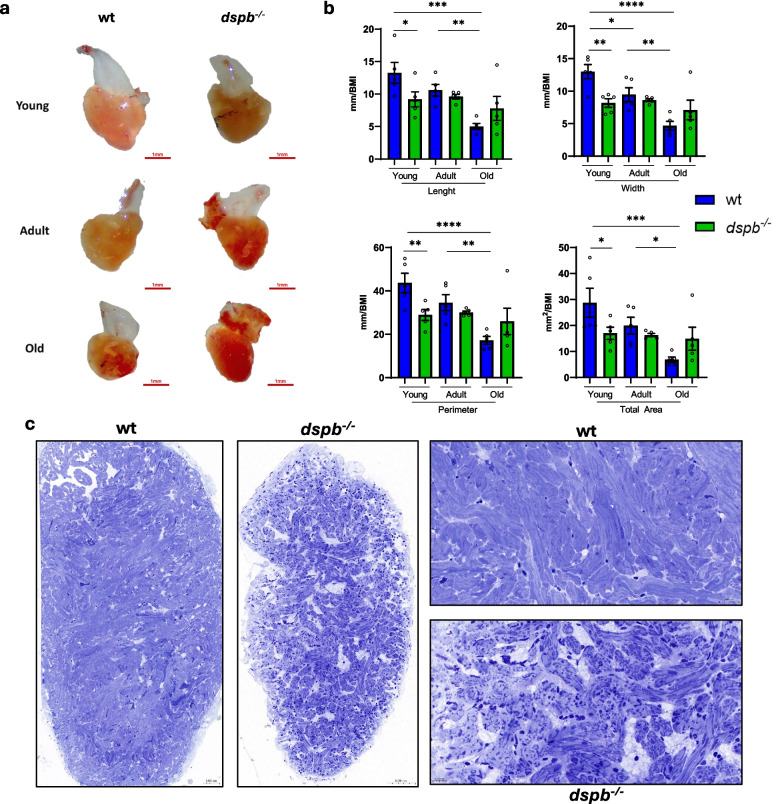
Macroscopical and histological study of zebrafish heart at different ages—Comparison between wt and *dspb*^*−/−*^. **a** Images of hearts after explantation were acquired with a Zeiss Stemi 305 Stereo Microscope and hearts were measured in length, width, perimeter and area. **b** Measurements show wt heart becoming smaller throughout time, while *dspb*^*−/−*^ heart being significantly smaller than wt heart at young age, with a tendency to a size increase at old age (*p < 0.05, **p < 0.01, ***p < 0.001, ****p < 0.0001) Sample size: *n* = 5/genotype and age group. Error bars: SEM. Test: Two-way ANOVA with Bonferroni correction. BMI: Body mass index. **c** Semi-thin sections stained with toluidine blue of adult zebrafish hearts from wt and *dspb*^*−*/−^ mutants, shown at low (vertical panels) and high magnification (horizontal panels). Mutant hearts show increased cellular density with numerous small, darkly stained nuclei and disorganized myocardial structure compared to wt controls

Macroscopic examination of explanted hearts revealed age-dependent changes in wt animals, as well as differences between genotypes. In the wt group, heart size decreased progressively with age. In contrast, *dspb*^*−/−*^ hearts were significantly smaller than wt hearts at juvenile stages in terms of length, width, area, and perimeter. These differences were no longer significant in adulthood, and in older animals, *dspb*^*−/−*^ hearts showed a trend to increased size compared with wt hearts (Fig. 5a-b; Table S4).

Histological analysis was performed to assess tissue composition, fatty infiltration, and signs of cardiomyocyte rarefaction or fibrosis. No differences in ventricular structure or cell rarefaction were observed between wt and *dspb*^*−/−*^ hearts at any stage (Table S4), and no signs of fibrotic replacement were detected (Fig. S4, last panels). Adipocyte infiltration was observed only at old age in both groups, with similar distribution and extent (Fig. S4, third panels). Semi-thin sections stained with toluidine blue revealed striking differences in cellular composition and myocardial organization between wt and *dspb*^*−/−*^ zebrafish hearts (Fig. 5c). At low magnification, *dspb*^−/−^ samples exhibited increased cellular density with numerous deeply stained nuclei. At higher magnification, wt myocardium showed well-organized sarcomeric structures and relatively sparse nuclei, whereas mutant myocardium displayed accumulation of rounded, densely packed nuclei and clear disorganization of the sarcomeric pattern. The increased nuclear density observed in *dspb*^*−/−*^ hearts likely reflects a combination of processes, including inflammatory cells infiltration (such as macrophages and neutrophils), fibroblast activation and proliferation, and potential apoptotic or regenerative activity associated with tissue remodeling.

**Fig. 6:**
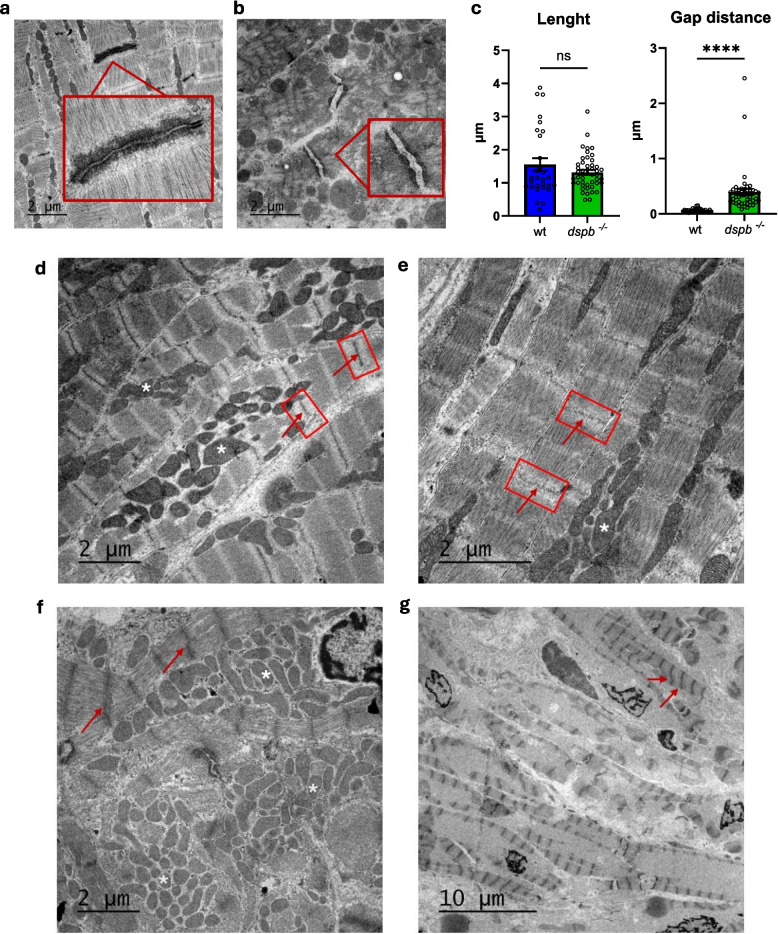
Structural differences in gap junctions and sarcomeres in cardiac tissue between wt and *dspb*^*−/−*^ mutants, observed by TEM. **a, b** Representative TEM images of gap junctions in wt (**a**) and *dspb*^*−/−*^**b** cardiac tissue. Insets highlight the ultrastructural differences. **c** Quantification of junction length (left) and intermembrane gap distance (right) in wt and *dspb*^−/−^ samples. While the total junction length remains comparable between genotypes, the intermembrane distance is significantly increased in *dspb*^−/−^ hearts, indicating defective cell–cell adhesion. Sample size: *n* = 47 for *dspb*^−/−^ and *n* = 29 for wt; Error bars: SEM. (****p < 0.0001, ns – not significant). **d, e** TEM images of wt cardiac tissue, showing well-aligned sarcomeres, clearly defined Z lines and homogeneously distributed mitochondria. The presence of intact I bands and regular intercalated discs reflects a preserved cardiac ultrastructure. **f, g** TEM images of *dspb*^−/−^ mutant cardiac tissue, highlighting sarcomere disorganization, loss of I band definition, and disrupted Z alignment, with thicker Z lines. Mitochondrial clustering and irregular intercalated discs are also evident, suggesting severe structural remodeling and impaired contractile function in *dspb*^−/−^ hearts. (red arrow – Z line; red rectangle – I band; white asterisk – mitochondria)

Gap junction integrity was compromised in *dspb*^*−/−*^ mutants. Representative TEM images of intercalated discs (Fig. 6a, b) highlight differences in cell–cell junction structure between wt and mutant cardiac tissue. In wt samples, gap and adherens junctions appear compact, with tightly apposed membranes and electron-dense plaques. In contrast, *dspb*^*−/−*^ mutants exhibit disrupted junctions with a significant increase in the intermembrane distance, suggesting altered mechanical coupling between cardiomyocytes. Insets in Fig. 6a-b provide magnified views of the junctional structures. Quantitative analysis of junctional parameters confirmed these findings (Fig. 6c). The mean junction length was similar between groups (wt 1.55 ± 0.19 µm vs. *dspb*^*−/−*^1.31 ± 0.07 µm), whereas intermembrane gap distance was significantly increased in *dspb*^*−/−*^ (0.39 ± 0.05 µm vs 0.06 ± 0.005 µm in wt, *p* < 0.0001), indicating a significant structural alteration. Furthermore, 90.6% (29/32) of junctions in wt samples were classified as normal, compared with only 9.6% (5/52) in *dspb*^−/−^ mutants, supporting a marked disruption of intercalated disc organization. TEM analysis of myocardial tissue also revealed striking differences in sarcomere organization between genotypes. Wt cardiomyocytes (Fig. 6d-e) displayed well-organized contractile apparatus, with clearly defined Z lines, aligned myofibrils, and distinct I-bands. Mitochondria were homogeneously distributed throughout the cytoplasm, and intercalated discs maintained a regular structure, indicative of a healthy, functional myocardium. In contrast, *dspb*^*−/−*^ samples (Fig. 6f-g) exhibited severe sarcomeric disorganization, characterized by loss of I band definition, misaligned Z lines, and irregular myofibril spacing. Mitochondria appeared abnormally clustered, and intercalated discs showed fragmented or irregular structures. Overall, *dspb*^*−/−*^ mutants exhibited marked disruption of cardiac ultrastructure, including altered sarcomeric organization and intercalated disc architecture.

Immunofluorescence analysis of desmosomal proteins revealed increased PKP2 integrated density and PKP2-positive area normalized to nuclear area in *dspb*^*–/–*^ hearts compared with wt, whereas the PKP2 intensity-to-nuclear-intensity ratio was not significantly different (Fig. S5). In contrast, plakoglobin displayed a significantly higher intensity-to-nuclear-intensity ratio in *dspb*^*–/–*^ myocardium, while integrated density and protein-positive area were comparable to wt. These findings indicate genotype-specific remodeling of desmosomal protein distribution, supporting the structural alterations in intercalated discs observed by TEM.

### Exercise interventions differentially improve cardiac function in dspb-/- adults

**Fig. 7:**
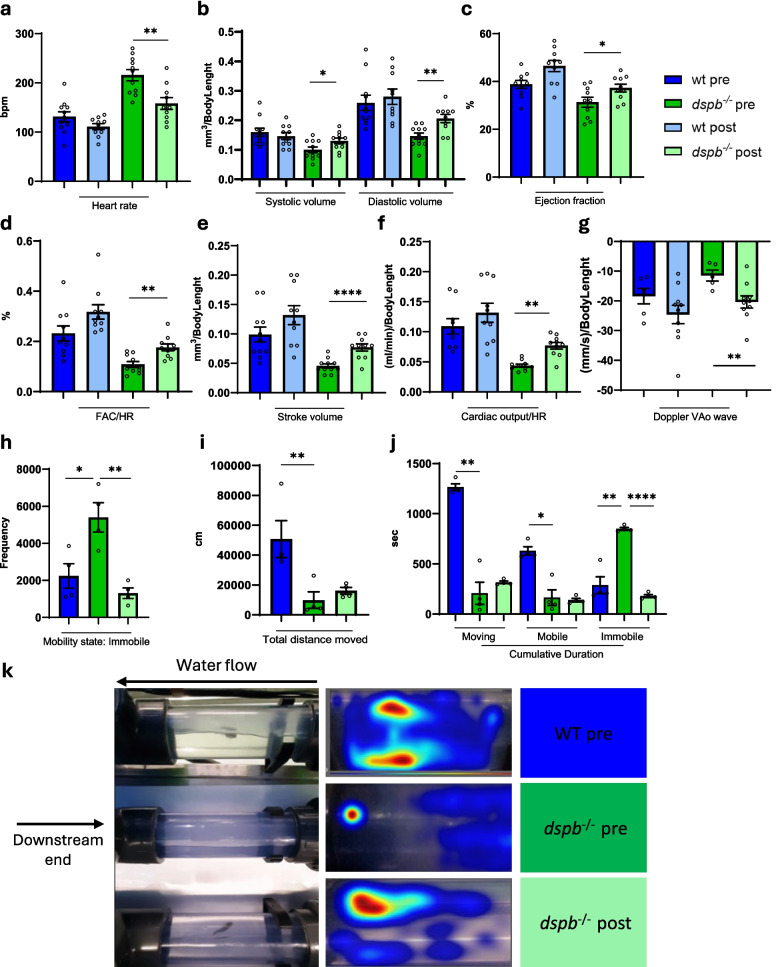
The impact of physical activity. **a**-**h** Results before and after long-time moderate training in *dspb*^−/−^ mutants, with significant differences, as mutants after one month show: **a** lower heart rate, normalization in **b** diastolic area, **c** systolic/diastolic volumes, **d** stroke volume, **e** cardiac output, **f** ejection fraction, **g.** fractional area change, but also in **(h)** aortic outflow (*p < 0.05, **p < 0.01, ***p < 0.001, ****p < 0.0001) Error bars: SEM. Sample size wt and *dspb*^*−/−*^ pre: *n* = 11; Sample size wt and *dspb*^*−/−*^ post: *n* = 10. Test: Two-way ANOVA with Bonferroni correction**. i-l** Endurance assays pre and post moderate training**.** Results show that *dspb*^*−/−*^ mutants were less able to cope with high intensity training compared to the wt group, but gradually increased endurance after a previous period of moderate training, as demonstrated by some parameters measured by EthoVision XT software (*p < 0.05, **p < 0.01, ***p < 0.001, ****p < 0.0001) Error bars: SEM. Sample size: *n* = 4. Test: Two-way ANOVA with Bonferroni correction. (l-top) Heat maps generated by EthoVision XT software that analyse movement during the endurance assays, showing how *dspb*^*−/−*^ mutants that have been previously trained during one month in the swimming tunnels have a better capacity of swimming against the water flow that *dspb*^*−/−*^ mutants that were not previously trained and therefore, trained *dspb*^*−/−*^ heat map is much more alike to the wt heat map. (l**-**bottom) Swimming tunnels used to analyse the impact of moderate training and endurance assays

One month of training in adult zebrafish (8 months) had a notable impact on cardiac parameters in *dspb*^*−/−*^ zebrafish. First, both groups showed 100% survival after the training period. In the *dspb*^*−/−*^ group, the heart rate significantly decreased after exercise compared to pre-exercise levels. The diastolic area increased markedly post-training, and both systolic and diastolic volumes increased in the *dspb*^*−/−*^ group. Additionally, stroke volume and cardiac output were also partially restored, along with ejection fraction and fractional area change. Aortic outflow parameters also improved. These findings suggest that sustained moderate exercise can positively modulate cardiac function and structure in *dspb*^*−/−*^ zebrafish (Table S3; Fig. 7a-h).

During endurance trials, *dspb*^*−/−*^ mutants exhibited reduced performance compared with wt group. Wt fish showed greater total distance travelled and higher cumulative movement duration than the *dspb*^*−/−*^ mutants. They also displayed longer periods of mobility and lower frequency of immobility. In contrast, mutants spent more time in immobile states, particularly at the end of the tunnel, indicating reduced resistance to flow (Table S5; Fig. 7i-k). Notably, *dspb*^−/−^ mutants subjected to one month of moderate training showed improved endurance compared with untrained mutants. The exercised *dspb*^*−/−*^ mutants exhibited reduced immobility frequency and shorter immobility duration (Table S5 and Fig. 7k). This improvement is further illustrated in Fig. 7l, where the heat map obtained from the EthoVision XT software shows that trained *dspb*^*−/−*^ mutants displayed a swimming pattern similar to wt fish. These findings support an improvement in endurance capacity after sustained moderate exercise in *dspb*^*−/−*^ mutants. In contrast, untrained mutants were unable to sustain swimming against the current and, as shown by the heat map, remained predominantly at the end of the tunnel, pushed away by the flow (Fig. 7l).

## Discussion

The role of physical activity in the onset and progression of ND-LVC, particularly in individuals carrying DSP truncating variants, remains uncertain [3]. In our cohort, carriers engaging in moderate to intense physical activity were diagnosed at a significantly younger age than sedentary individuals. However, this earlier diagnosis was not associated with increased severity of structural or arrhythmic abnormalities, nor with a higher incidence of adverse cardiovascular events. This uncertainty has also been highlighted by Martínez-Solé et al. [3], who reported that although high-intensity exercise may be harmful in classical forms of ACM, its impact remains controversial and less well defined in non-classical phenotypes, including those involving DSP truncating variants [11]. One possible explanation is that physically active individuals undergo more frequent medical evaluations, leading to earlier diagnosis, whereas sedentary carriers may remain undiagnosed for longer periods. Alternatively, the earlier diagnosis and milder phenotype observed in active individuals may reflect a modulatory rather than deleterious effect of exercise. To further explore this hypothesis, we employed a *dspb*^*−/−*^ zebrafish model engineered to carry a truncating mutation homologous to the human *DSP* p.Q447* variant. This variant exhibits high penetrance and has been well-documented in regional cohorts [2, 12]. This model allowed us to explore disease mechanisms and the effects of exercise under controlled experimental conditions.

Using this model, we identified developmental, functional, and structural abnormalities consistent with ND-LVC. Mutant embryos exhibited reduced viability and developmental delay, characterized by shorter body length and enlarged yolk sacs, indicating impaired embryogenesis. Similar findings were reported by Asimaki et al. using a transgenic zebrafish model of ACM carrying a plakoglobin mutation (2057del2), in which mutant embryos showed a markedly reduced survival rate of 45%, compared with 77% in controls [13].

Among *dspb*^*−/−*^ larvae surviving beyond early embryogenesis, clear functional alterations were evident. Most notably, mutants exhibited tachycardia as early as 3 dpf, with persistently elevated heart rate compared with wt controls. This may represent a compensatory response to reduced stroke volume, helping to maintain cardiac output. Increased respiratory rate was also observed, further supporting a heart failure-like phenotype. Interestingly, this differs from previous zebrafish models of *DSP* and *PKP2* deficiency, which reported bradycardia rather than tachycardia [13, 14]. In adult zebrafish, echocardiographic analyses confirmed systolic and diastolic dysfunction. Our DSP model aligns more closely with ND-LVC than with classical ACM, as fibrosis was not observed. Ventricular size was reduced in *dspb*^*−/−*^ zebrafish at young adult stages, although smaller hearts are not typically described in human DSP cardiomyopathy. However, compensatory ventricular enlargement is also uncommon in carriers of *DSP* nonsense mutations. Clinically, *DSP*-related cardiomyopathy in humans is generally classified as left-dominant ACM [2] or within the recently coined ND-LVC phenotypic classification [1]. Reduced stroke volume likely contributes to decreased cardiac output and may trigger compensatory tachycardia to preserve cardiac performance.

Beyond the observed functional phenotype, our findings indicate that *dspb* loss is partially compensated by upregulation of desmosomal genes such as *dspa* and *pkp2,* supporting compensatory mechanisms among desmosomal components, consistent with previous studies [15–18]. Nonetheless, this transcriptional adjustment appears insufficient to maintain proper signaling balance and does not prevent disruption of key cardiac signaling pathways, including Wnt/β-catenin, TGF-β, and Hippo/YAP-TAZ.

In the Wnt/β-catenin pathway, early downregulation of *ccnd1* and *myc* at 6 dpf in *dspb*^−/−^ larvae suggests impaired cardiomyocyte proliferation during early development. Interestingly, this trend reverses at 20 dpf with transient upregulation, possibly reflecting a compensatory effort to restore proliferative capacity. Nevertheless, in adult stages, both genes remain consistently downregulated, indicating a sustained reduction in Wnt signaling that likely contributes to impaired cardiac regeneration and structural abnormalities observed in mutant hearts. These findings are consistent with the stage-specific regulatory function of Wnt signaling in cardiac biology [19, 20].

Regarding TGF-β signaling, increased expression of *smad2* and *smad3* at 20 dpf in *dspb* mutants may represent an early fibrotic or remodeling response to tissue stress, particularly in the developing heart. In adults, only partial upregulation of *smad3* persists, suggesting a chronic low-grade activation of fibrotic pathways. This may contribute to the ultrastructural desmosomal alterations observed by TEM and aligns with the known role of TGF-β in pathological cardiac remodeling [21]. The Hippo/YAP-TAZ pathway also displayed distinct temporal dysregulation: *ccn2a* was suppressed at 6 dpf but upregulated at 20 dpf, while *ccn2b* remained consistently downregulated across all stages. These results suggest a failure to properly activate extracellular matrix remodeling signals during early development, followed by a delayed compensatory response. The persistent repression of *ccn2b*, which plays a key role in cardiac regeneration, further supports impaired tissue recovery and maintenance [22, 23].

Together, these findings emphasize that while desmosomal gene redundancy may buffer the structural impact of *dspb* loss to some extent, the disruption of downstream signaling is robust and persistent. The stage-specific alterations in gene expression patterns reinforce the idea that *dspb* is essential for integrating structural and signaling networks during heart development. These patterns are in agreement with those observed in other zebrafish models of ACM, underscoring the relevance of this model to human cardiac disease [14, 16, 24].

Emerging evidence indicates that desmosomal dysfunction affects not only structural integrity but also intracellular signaling networks. Consistent with previous ACM models, dspb deficiency was associated with dysregulation of Wnt/β-catenin, Hippo/YAP-TAZ, and TGF-β pathways, which are involved in cardiomyocyte proliferation, fibrosis, and pathological remodeling. These findings further support the interplay between desmosomal disruption and maladaptive signaling mechanisms in ACM phenotypes [14–16, 22–24].

In addition to the functional alterations, structural and ultrastructural analyses revealed important myocardial abnormalities in dspb-/- hearts. Histological evaluation of adult hearts revealed no major differences in myocardial architecture between *dspb*^*−/−*^ and wt fish. The distribution of cardiomyocytes appeared preserved, and no signs of fibrotic replacement were observed. Adipocyte infiltration, often associated with advanced cardiomyopathy, was present in both groups at older stages, with similar localization and extension. This contrasts with findings in other *dspb* mutant models that report cardiomyocyte rarefaction, vascular dilation, and pronounced adipogenesis at later stages [14].

Toluidine blue staining revealed increased nuclear density and altered cellular composition in *dspb*^*–/–*^ hearts, consistent with non-myocyte expansion. This may reflect early fibrotic or inflammatory remodeling, in line with human DSP-cardiomyopathy, where myocarditis-like episodes are reported in up to 20% of patients, which are linked to disease progression and arrhythmias [25].

Wt cardiomyocytes displayed well-organized sarcomeres with clearly defined Z-lines and intact I-bands. In contrast, dspb mutants exhibited severe sarcomere disorganization, with hyperintense Z lines and a notable absence of I bands, suggesting myofibrillar instability. Similar results were observed in a study by Song et al. in zebrafish models carrying a mutation in Pr72, a negative regulator of the Wnt signaling pathway, which also showed disrupted sarcomere architecture and loss of I-bands [26]. Additionally, disorganized and delocalized desmosomes have also been observed in Celeghin’s desmoplakin model [14]. Wt cardiomyocytes exhibited tightly apposed intercalated discs with intact desmosomes and gap junctions. However, *dspb*^*−/−*^ mutants displayed disrupted intercalated discs, characterized by widened intercellular spaces and fragmented desmosomes, consistent with defective cell–cell adhesion and impaired junctional integrity. These findings are in line with those of Celeghin et al., who reported similar intercellular widening in desmoplakin-deficient zebrafish [14]. Interestingly, ultrastructural analyses of human ACM samples by Basso et al. corroborates this phenotype, revealing a widened fascia adherens gap in patients compared to controls [27]. Moreover, in wt samples, mitochondria were uniformly distributed, typically surrounding sarcomeres. In the *dspb*^*−/−*^ samples, mitochondria appeared clustered around disorganized sarcomeres, potentially as an adaptive response to energy demand fluctuations.

Considering the controversial role of exercise in the clinical setting, we evaluated its impact in adult *dspb*^*−/−*^ zebrafish. After one month of moderate training, several cardiac functional parameters were partially restored toward wt levels. Similarly, endurance assays showed that trained *dspb*^*−/−*^ mutants exhibited improved performance compared with untrained mutants, with mobility patterns approaching those of wild-type fish. These findings suggest that moderate exercise may enhance both cardiac function and endurance capacity. In contrast, untrained mutants displayed reduced mobility and impaired ability to swim against water flow, spending most of the time at the downstream end of the tunnel. Importantly, survival remained 100% in both groups following both long-term and high-intensity training.

To our knowledge, cardiac function has not been previously evaluated in adult zebrafish models of DSP deficiency after prolonged exercise. While one prior study examined the effects of mild training in larval zebrafish using increased medium viscosity, that protocol was associated with higher mortality in trained versus untrained groups [14]. Our findings therefore provide novel evidence that moderate exercise may confer functional benefits in adult desmosomal cardiomyopathy models, without exacerbating mortality risk.

In recent years, three principal zebrafish models of *dsp* deficiency have been developed, each contributing complementary insights into the role of desmoplakin in cardiac development and disease. The morpholino-based model by Giuliodori et al. (2018) revealed early structural and functional cardiac defects [16], while the stable CRISPR-generated mutants by Celeghin et al. (2023) and our own *dspb*^*−/−*^ mutant line recapitulate distinct features of desmoplakin cardiomyopathy in a longitudinal manner [14]. Together, these models underscore the versatility and translational relevance of zebrafish for investigating desmosomal dysfunction in inherited cardiomyopathies.

Our *dspb*^*−/−*^ model offers several notable strengths, including longitudinal characterization from early larval stages to adulthood, as well as comprehensive assessment of cardiac function, molecular signaling, structural remodeling, survival, and exercise response. A comparative timeline (Table S6) summarizes the experimental designs, life stages, and phenotypic outcomes assessed in each model, illustrating the zebrafish's capacity to interrogate disease mechanisms at molecular, structural, and functional levels throughout its lifespan.

However, several limitations should also be acknowledged, including incomplete replication of some human phenotypes, such as fibrosis and arrhythmia, differences in cardiac physiology between zebrafish and human hearts (two-chambered vs four-chambered heart), and the absence of therapeutic intervention studies to date. Despite these limitations, our findings, together with previous zebrafish models, underscore the utility of zebrafish as a powerful in vivo platform for mechanistic research and preclinical evaluation of therapeutic strategies in desmosomal cardiomyopathies.

Several limitations of the human study should also be acknowledged. The rarity of DSP-related cardiomyopathy resulted in a relatively small sample size, which limited the complexity of multivariable analyses and precluded reliable subgroup analyses based on type of sport, family clustering, or detailed genotype stratification for age at diagnosis and event-free survival. One could argue that physically active individuals might have had easier access to medical examinations, potentially influencing age at diagnosis. However, no preparticipation screening program for athletes was in place in our region during the study period that could explain differential access to ECG or echocardiographic evaluation.

## Conclusions

Our *dspb*^*−/−*^ zebrafish model exhibited a characteristic ND-LVC phenotype, including baseline systolic dysfunction, reduced stroke volume, and compensatory tachycardia. Ventricular size was also reduced, reflecting impaired cardiac growth and limited contractile reserve. The phenotype evolved with age, showing dynamic progression throughout the zebrafish lifespan, and was partially normalized by moderate physical training in adults, which also enhanced endurance capacity in mutants. At the structural level, desmoplakin deficiency leads to sarcomeric disarray and junctional remodeling, providing insights into the pathophysiology of DSP-related cardiomyopathies. These changes were accompanied by significant transcriptional alterations in key cardiac signaling pathways, including Wnt, TGF-β, and Hippo.

These findings reinforce the zebrafish as a powerful translational model for exploring genotype-specific responses to lifestyle interventions in inherited cardiomyopathies and support moderate exercise as a potentially safe and beneficial intervention in selected *DSP* variant carriers.

## Materials and methods

### Study design and endpoints

Based on the predefined objectives of the study, endpoints were classified as primary (pre-specified) or exploratory to limit type-I error arising from multiple comparisons.

Primary endpoints were predefined as follows:

1. Human cohort: Age at clinical diagnosis of DSP truncating variant carriers and event-free survival from MACE.
2. Zebrafish model: Adult cardiac function, assessed by echocardiography (ejection fraction and fractional area change). Cardiac output and stroke volume in adult zebrafish. Survival in *dspb*^*–/–*^ versus wt zebrafish. Exercise-induced change in cardiac function (pre- vs post-training echocardiographic assessment).

All other variables were considered exploratory endpoints, including: Additional echocardiographic parameters (ventricular areas, volumes, Doppler-derived indices, heart rate, respiratory frequency). Embryonic morphology and larval heart rate measurements. Endurance and swimming performance parameters. Histological measurements (macroscopic heart dimensions, cellular density). Immunofluorescence-based quantification of desmosomal proteins. Ultrastructural TEM measurements (junctional length, intermembrane gap distance). Gene expression analyses of desmosomal components and signaling pathway markers. Exploratory analyses were performed to generate mechanistic hypotheses and were interpreted descriptively.

### Clinical phenotype evaluation, genetic study and physical activity assessment

Seventy-five patients from 19 families carrying truncating variants in the *DSP* gene (45 affected and 30 unaffected) identified at the Inherited Cardiac Diseases Unit of the Hospital Universitario Virgen de la Arrixaca were included in the study. Clinical data were systematically reviewed and included personal and family medical history, 12-lead electrocardiogram (ECG), Holter-ECG and cardiac imaging. Genomic DNA was extracted from peripheral blood samples, and genetic testing was performed as part of the standard diagnostic protocol for inherited cardiomyopathies.

ND-LVC was defined following the 2023 ESC Cardiomyopathy Guidelines as the presence of left-ventricular systolic dysfunction (LVEF < 50%) and/or late gadolinium enhancement (LGE) in the absence of LV dilatation. LV dilatation was determined using the Henry index, where LV end-diastolic diameter (LVEDD) is normalized to body size and age using the formula [28]: 

$$Henry\%=100 \times (LVEDD/((45.3 \times (BSA)^{1/3})-(0.03 \times age)- 7.2))$$

A Henry index > 112% was considered indicative of pathological LV dilatation and therefore consistent with DCM, whereas values ≤ 112% indicated the absence of dilatation. Accordingly, patients with LVEF < 50% and/or positive LGE with Henry% ≤ 112% were classified as ND-LVC, while those with Henry% > 112% were considered DCM. Quantification of LGE on CMR was performed using a > 5-SD threshold above remote myocardium and expressed as % of LV mass. LGE > 15% of LV mass or a characteristic subepicardial/mid-wall pattern was considered significant. Definitions of major adverse cardiac events (MACE) followed ESC 2023 guidelines and included sustained ventricular arrhythmia, resuscitated cardiac arrest, or cardiac death. All adjudications were performed blinded to physical activity status. Information on physical activity was collected prospectively. Data were verified through structured telephone interviews using a standardized questionnaire. Participants were asked to report the type, frequency, and duration of physical activity performed during a “typical week” in the 3 years preceding the clinical diagnosis [29]. Only activities estimated to raise heart rate above 70% of the age-predicted maximum heart rate were considered.

Moderate physical activity level was defined between > 2 and < 5 h per week, and intense physical activity was defined as > 5 h per week. To facilitate comparisons ensuring enough cases, those who were sedentary or engaged in light physical activity were grouped as sedentary/light exercise and those who were engaged in more than 2 h per week were grouped as moderate/intense exercise. The type of sport was classified according to the EAPC classification as "skill", "power", "mixed", and "endurance" [30]. For statistical analysis, "skill" and "power" were grouped together, and "mixed" and "endurance" were grouped together.

All procedures were conducted in accordance with the Declaration of Helsinki and institutional ethical standards. The study protocol was reviewed and approved by the Ethics Committee of the Virgen de la Arrixaca University Hospital (reference number 2023–1–12-HCUVA). Written informed consent was obtained from all participants prior to inclusion in the study.

### Care and maintenance of zebrafish

Zebrafish (*Danio rerio*) were obtained from the Zebrafish International Resource Center and were maintained according to standard procedures [31]. Water quality parameters were constantly measured with an automated system [32]. Animal handling and experimentation followed the protocols and guidelines established by the European Union Directive 2010/63/EU and the Spanish RD 53/2013. These protocols were approved by the Animal Experimentation Ethics Committee (CEEA) of the Virgen de la Arrixaca University Hospital-IMIB (approval number Nº A13211204, REGA ES300303340098).

### Zebrafish model generation and experimental design

A zebrafish *dspb*^*−/−*^ mutant line carrying a truncating variant (p.T449fs*) was generated using CRISPR/Cas9 genome editing to model the human DSP p.Q447* variant. Homozygous mutants and wild-type controls were analyzed across developmental stages. Cardiac function was assessed in larvae and adult fish, and structural, molecular, and survival phenotypes were evaluated. Detailed experimental procedures are provided in the Supplementary Material.

### Functional, structural, and molecular analyses

Cardiac function was assessed using microscopy-based heart rate measurements in larvae and echocardiography in adult zebrafish. Structural characterization included histological and ultrastructural analyses. Gene expression of desmosomal components and key signaling pathways was evaluated using qPCR. Detailed experimental procedures are provided in the Supplementary Material.

### Swimming capacity test

**Fig. 8:**
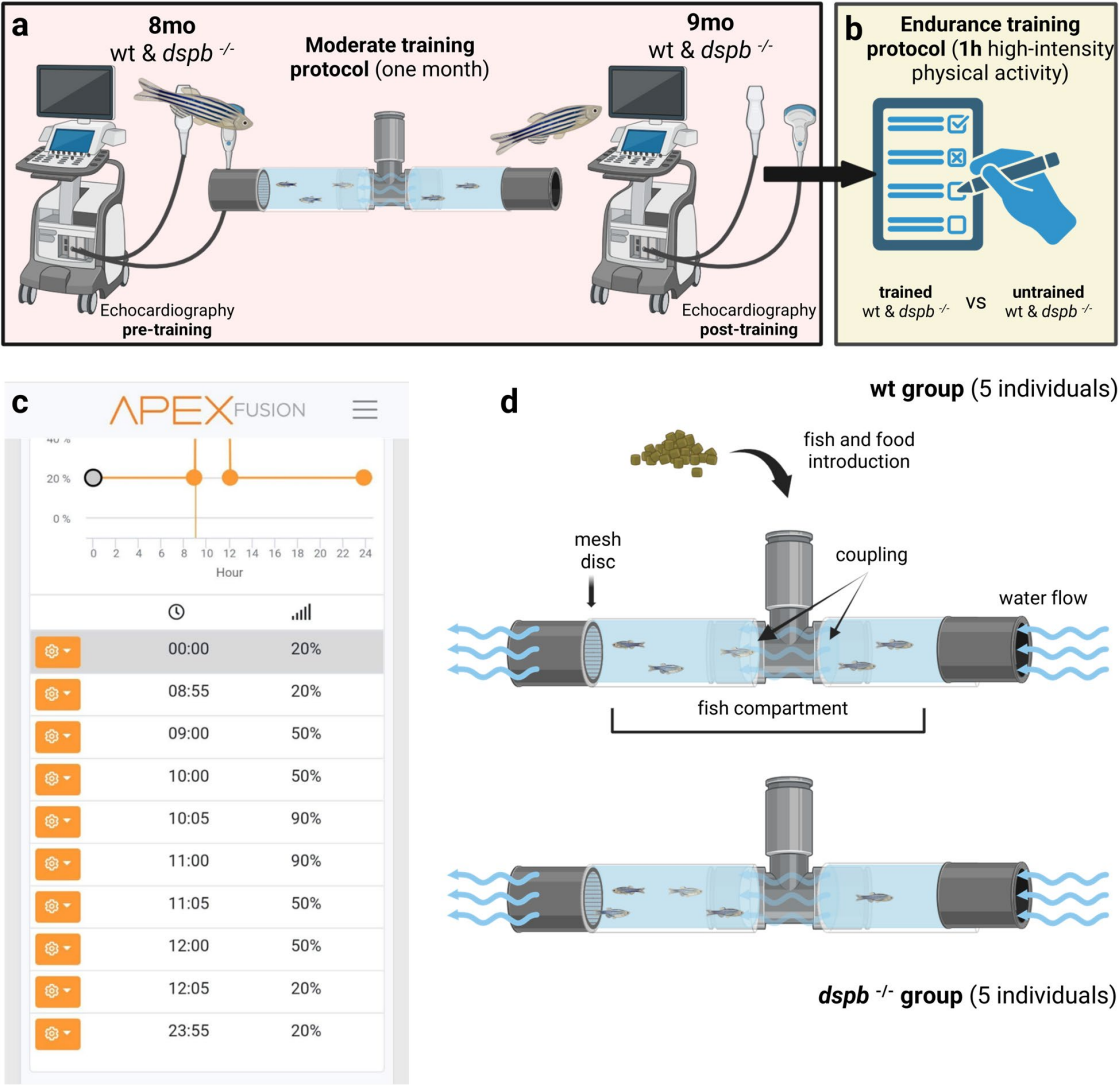
Swimming protocols explained. **a** Moderate one-month training schedule, with echocardiography pre and post training protocol. **b** High-intensity endurance training protocol for previously trained and untrained fish. **c** Moderate one-month training schedule from day 2 to day 30, showing daily variations in flow intensity as part of the exercise regimen. **d** The fish were divided into two groups and placed in the swim tunnels for one month. All the fish underwent the same training simultaneously with groups of 5 individuals per tunnel. Figure created in BioRender

To evaluate the impact of swimming performance on the mutant phenotype, animals were placed in a swim tunnel (Fig. 8d). Swimming performance was assessed using a custom-built swim tunnel system (dimensions: 61 cm length ×10 cm internal diameter). Each tunnel was directly connected to the main zebrafish facility water circulation, ensuring identical temperature (28.0 °C± 0.5 °C), pH, conductivity, dissolved oxygen, and 14:10 h light/dark photoperiod. All environmental variables were continuously monitored and logged using Apex Fusion® (Neptune Systems). Adult zebrafish (mixed sex) were tested in groups of five fish per tunnel. Wild-type and *dspb*^*−/−*^ animals were always placed in separate tunnels, and all groups completed the same flow-controlled training protocol under identical conditions. Fish were allowed to acclimate for 24 h at a low flow speed (3 cm s⁻^1^, 20% flow intensity). After this acclimation period, the fish underwent a one-month training regimen for four weeks (Fig. 8a and c), followed by a final endurance test lasting one hour (Fig. 8b). The water flow was created using an electric pump and intensity flow was controlled using APEX Fusion software (https://apexfusion.com/). [32] The one-month training protocol involved different stages of swimming, during which the water velocity gradually increased. In the first stage, the velocity was raised to 5.5 cm s⁻^1^ over 5 min (50% intensity). The second stage involved maximum intensity swimming at 10 cm s⁻^1^ (90% intensity). In the third stage, the flow was gradually decreased to 5.5 cm s⁻^1^ (50% intensity). For the rest of the time, the fish swam in the tunnel with a basal water flow of 3 cm s⁻^1^ (20% intensity).

### Endurance training protocol

To assess endurance capacity, fish were subjected to a resistance test, with groups of fish that had never been exposed to swimming tunnels serving as controls. This experiment involved placing each individual fish, both *dspb*^*−/−*^ and control, whether previously trained or not, into a swimming tunnel for one hour at maximum intensity flow at 12 cm s⁻^1^ (100%). The endurance of groups that had undergone a month swimming training in the tunnel was compared to that of groups with no prior swimming sessions (Fig. 8b).

The evaluation of swimming endurance was conducted using the EthoVision XT software (Noldus) [33]. This software analyses video recordings of fish swimming in the tunnels, detects motion, generates heat maps, and collects various mobility-related data. Parameters measured by the software include total distance travelled (cm), cumulative duration of moving or stationary states (seconds), frequency, and cumulative duration of different mobility states. These parameters provided a comprehensive assessment of the fish's endurance capabilities under the given experimental conditions.

### Statistical analysis

All statistical analysis were performed using GraphPad Prism 10 software (GraphPad Software Inc., San Diego, CA, USA). The results were expressed as mean ± standard error of the mean (SEM), unless otherwise stated. Normality was assessed using the Shapiro–Wilk test. For normally distributed quantitative variables, comparisons between two groups were performed using Student’s *t*-test, and comparisons involving more than two groups were analyzed using two-way ANOVA followed by Bonferroni post-hoc correction. When normality assumptions were not met, Mann–Whitney U tests (two groups) or Kruskal–Wallis test (more than two groups) were applied. Survival analyses were performed using Kaplan–Meier estimates and Cox proportional hazards regression models. Group differences in survival curves were assessed using the log-rank test. Multivariable Cox proportional hazards models were used to estimate adjusted hazard ratios (HRs) with 95% confidence intervals, accounting for potential confounders. Covariates included sex and physical activity status (moderate/intense vs sedentary/light). Differences were considered statistically significant at *p* < 0.05 (*), *p* < 0.01 (**), *p* < 0.001 (***) and *p* < 0.0001 (****) and ns (not significant) for *p* > 0.05.

## Supplementary Information

Supplementary Material 1.

## Data Availability

All data generated or analysed during this study are included in this published article [and its supplementary information files].
