# Supplementary material for: Zebrafish dspb-/- mutant as a model for non-dilated left ventricular cardiomyopathy: exploring cardiac dysfunction and exercise modulation
Source: Mol Biomed. 2026 Jun 15;7:90. doi: 10.1186/s43556-026-00476-7 (PMC13269591; doi:10.1186/s43556-026-00476-7)
Supplement: Supplementary file 1 — Supplementary Material 1. [file 43556_2026_476_MOESM1_ESM.docx]

**Zebrafish *dspb^-/-^* Mutant as a Model for Non-Dilated Left Ventricular Cardiomyopathy: Exploring Cardiac Dysfunction and Exercise Modulation**

**Authors**

Serena Munteanu¹, Jesús Wagih Gómez¹, Ángel Júdez Serrano², Daniel Saura Espín³, Juan José Santos Mateo³, María Elisa Nicolás Rocamora¹, Cristina Gil Ortuño², Ángel Bernabé García², Juan Ramon Gimeno Blanes⁴, María Luisa Cayuela Fuentes⁵, María Sabater Molina⁶

^1^ Cardiogenetic Laboratory. Biomedical Research Institute of Murcia (IMIB), Murcia, Spain. University of Murcia

^2^ Cardiogenetic Laboratory. Biomedical Research Institute of Murcia (IMIB), Murcia, Spain. University of Murcia

^3^ Inherited Cardiac Disease Unit (CSUR), Virgen de la Arrixaca University Hospital, Murcia, Spain

^4^ Inherited Cardiac Disease Unit (CSUR), Virgen de la Arrixaca University Hospital, Murcia, Spain. European Reference Network for Rare and Low Prevalence Complex Diseases of the Heart (ERN-Guard Heart), Amsterdam, The Netherlands. Department of Internal Medicine (Cardiology), University of Murcia, Murcia, Spain; Cardiogenetic Laboratory. Biomedical Research Institute of Murcia (IMIB), Murcia, Spain

^5^ Biomedical Research Institute of Murcia (IMIB), Murcia, Spain. General Surgery Department, Virgen de la Arrixaca University Hospital, Murcia, Spain

^6^ Department of Legal and Forensic Medicine, Faculty of Medicine, University of Murcia, 30110, Murcia, Spain. Cardiogenetic Laboratory, Biomedical Research Institute of Murcia (IMIB), Murcia, Spain. European Reference Network for Rare and Low Prevalence Complex Diseases of the Heart (ERN-Guard Heart), Amsterdam, The Netherlands; Cardiogenetic Laboratory. Biomedical Research Institute of Murcia (IMIB), Murcia, Spain

**Funding**

The authors acknowledge the support of the Instituto de Salud Carlos III (ISCIII) through the project PI18/01231, co-funded by the European Union (European Regional Development Fund – ERDF, ‘A way to make Europe’).

**Acknowledgments**

We thank the patients who participated in this study for their invaluable contribution. We also acknowledge Dr. Rubén Corral, Dr. Diana García and Dr. Eva Cabrera for their assistance with the experiments.

**Detailed experimental procedures**

## *Generation of Zebrafish mutant lines through CRISPR/Cas9*

To model the human p.Q447* (NM_004415.3: c.1339C>T) *DSP* variant, we generated a zebrafish mutant line for desmoplakin b *(dspb)*. This variant is a founder nonsense variant in patients from our region [2, 14] and was selected it for modelling in zebrafish using the CRISPR/Cas9 approach. To delete the functional protein, a single guide RNA (sgRNA) was synthetised using CRISPR RNA (crRNA: GGCCACTTACAGCGCTGGAC) for zebrafish *dspb* and tracrRNA (trans-activating crRNA; catalogue no. 1072533), both purchased from Integrated DNA Technologies (IDT). Exon 10 was selected based on sequence homology between human DSP and zebrafish *dspb* (ClustalW alignment) ([www.ebi.ac.uk/Tools/msa/clustalw2](file:///C:\Users\Serena\OneDrive%20-%20UNIVERSIDAD%20DE%20MURCIA\Desktop\www.ebi.ac.uk\Tools\msa\clustalw2)). sgRNA design and off-target prediction were performed using CHOPCHOP v3 ([www.chopchop.cbu.uib.no](file:///C:\Users\Serena\OneDrive%20-%20UNIVERSIDAD%20DE%20MURCIA\Desktop\www.chopchop.cbu.uib.no)) (Table S7). The selected guide showed no predicted off-target loci with 1, 2, or 3 mismatches across the zebrafish genome (MM1–MM3 = 0). Therefore, no empirical sequencing of off-target sites was required, in line with current zebrafish CRISPR guidelines.

The gRNA was mixed with the Cas9 nuclease (IDT, catalog no. 1081058). The prepared mix was microinjected into the yolk of one-cell-stage embryos using a microinjector (Narishige) (0.5 to 1 nL per embryo). The same amounts of gRNA were used in all the experimental groups. The efficiency of gRNA was checked by amplifying and Sanger sequencing the target sequence with a specific pair of primers (Table S7).

F0 injected embryos were genotyped to verify if the cut was produced. Stable mutants were generated by outcrossing F0 injected fish adults with wildtype (wt) fish to select F1 founders. To ensure removal of any low-probability CRISPR-independent background variants, we performed three generations of outcrossing (F1–F3) before establishing experimental cohorts. All animals used in phenotyping experiments (*wt*, *dspb^+/–^*, *dspb^–/–^*) were generated from F3 heterozygous intercrosses, ensuring a uniform, outcrossed genetic background.

## *Genomic DNA extraction and genotyping of dspb mutant lines*

For genotyping fish, genomic DNA extraction was performed from tail fin clipping. Fish were previously anesthetized dissolving 0.168 mg/mL tricaine in Egg Water medium, then placed over a clean Petri dish and a piece of the tail fin was cut and placed in an Eppendorf tube in Lysis Buffer (50Mm NaOH). Fish were then placed in separate aquariums until genotyping protocol was finished, and results were analysed. After genotype was confirmed, fish were grouped and placed in freshwater aquariums. Genotyping of *dspb* mutant line was performed by Sanger direct sequencing of *dspb*-specific PCR products.

## *Viability assays*

Viability assays have been conducted by counting live and dead larvae every two days for a month, to calculate the survival percentage and obtain survival curves which were compared using GraphPad Prism survival analysis. Wt embryos derived from wt adults crossed were used as control group.

## *Body measurements*

Embryos at 3 days post-fertilization (dpf) were anesthetized by dissolving 0.168 mg/mL tricaine buffered in Egg Water medium at room temperature, then positioned laterally in Petri dish and images were acquired using a Zeiss Stemi 305 Stereo Microscope with an integrated 1.2 Megapixel camera. Images were analysed using the Zeiss Labscope 4.2.1 software, measuring the body length, eye size, cardiac region size and determining the percentage of control or mutant embryos with morphological alterations or developmental delay.

## *Cardiac function study*

Cardiac function was analysed in zebrafish embryos at 3 dpf using a Zeiss Stemi 305 Stereo Microscope. Zebrafish embryos were treated with 1-phenyl 2-thiourea (PTU) from 1 dpf to delay pigment formation. Prior to the study at 3 dpf, embryos were anesthetized with tricaine (168 mg/mL) buffered in Egg Water medium at room temperature, with the embryo positioned laterally in Petri dish. Embryos were immersed for **<**2 min before acquisition, and heart rate was evaluated by recording for 15 seconds and extrapolating beats per minute (bpm). The mean heart rate of *dspb^-/-^* was compared to the wt control group. All conditions (tricaine concentration, exposure duration, temperature and lighting) were identical for wt and *dspb^–/–^* embryos, ensuring that any anesthesia-related heart rate modulation equally affected both groups.

Underwater echocardiography was performed in adult zebrafish using a Vevo LAZR-X echocardiography station (FujiFilm VisualSonics) equipped with a high frequency transducer (MX700, 50MHz). Fish were anesthetized with tricaine (168 mg/mL) and placed ventral side up on acetate bed designed for this purpose. One dimensional (M-Mode) and two-dimensional (B-Mode) images, color Doppler and pulsed-wave Doppler signals were recorded in long axis (PSLAX) and short axis views, with the probe parallel and perpendicular, respectively, to the long axis of the fish. M-Mode images were used to determine respiratory frequency, while B-Mode images were used to obtain data regarding ventricular area and volume during both systole and diastole. Pulsed wave Doppler images were optimized for either atrioventricular inflow or ventricular outflow assessment. To account for heart rate variation in ventricular measurements, at least three B-mode image sequences containing ≥3 cardiac cycles were recorded. For both pulsed wave Doppler and color Doppler, ≥5 s image sequences including ≥3 cardiac cycles were recorded. After image acquisition, fish recovered in aerated free water. Image analysis was performed using the Vevo Lab software (version 3.1.1, FujiFilm VisualSonics) focusing on the cardiac package in order to obtain parameters such as: ejection fraction (%), cardiac frequency (beats/min) and respiratory frequency (breaths/min), systolic and diastolic area (μm^2^) and volume (mm^3^), distance from apex to ventricle opening (mm), ventricular entry diameter (mm) and Doppler A and E waves velocity (mm/s) for atrial contraction and early diastole. All these parameters were measured in 11 individuals/group (*dspb^-/-^* mutants and wt group) pre-training and in 10 individuals/group (*dspb^-/-^* mutants and wt group) post training. All echocardiographic measurements and analyses were performed under blinded conditions.

## *Histological studies*

Five individuals were selected for both wt and *dspb*^-/-^ groups at three different ages: young (10 months old), adult (1.5 years old) and old (2.5 years old). Following explantation, adult zebrafish hearts were placed on a Petri dish in a PBS droplet and images were acquired using a Zeiss Stemi 305 Stereo Microscope with an integrated 1.2 Megapixel camera. Macroscopic measurements, including heart length, width, perimeter, and total area, were obtained using Zeiss Labscope v4.2.1 software.

After imaging, hearts were fixed in 4% paraformaldehyde for 24h at 4^o^C, then washed and stored in 70% ethanol at 4^o^C prior to paraffin embedding. Serial 3 μm sections were obtained and stained with Hematoxylin/Eosin for general histological assessment. To evaluate myocardial fibrosis, adult zebrafish hearts were stained using Masson’s Trichrome, which differentially stains collagen fibers. Sections were digitalized using the 3DHISTECH Pannoramic MIDI II slide scanner, while image analysis was performed using the QuPath version v0.5.1.0 software, determining the percentage of the total tissue area and fatty tissue, and to establish if there is any disruption of cellular adhesion or presence of fibrosis.

*Immunofluorescence staining and image analysis*

Immunofluorescence was performed on 10 μm paraffin-embedded zebrafish heart sections mounted on lysine-coated slides. Sections were incubated at 50 °C for 1 h, followed by deparaffinization and rehydration through graded xylene and ethanol series. Antigen retrieval was carried out for 30 min at 95 °C in a basic retrieval buffer. After cooling, sections were rinsed in PBS and blocked for 1 h at room temperature with blocking solution (3% BSA, 0.1% Tween-20 in PBS).

Primary antibodies diluted in blocking solution were applied and incubated for 2 h at room temperature or overnight at 4 °C in a humidified chamber. The following antibodies were used: anti-plakoglobin (mouse monoclonal, clone PG5.1; Progen, cat. no. 61005) and anti-plakophilin-2 (rabbit polyclonal; Prestige Antibodies®, Sigma-Aldrich, cat. no. HPA014314). After washing in PBS-T (PBS with 0.1% Tween-20), sections were incubated for 1 h in the dark with appropriate fluorescent secondary antibodies and Hoechst for nuclear counterstaining. Slides were mounted using an aqueous mounting medium.

Fluorescence images were acquired using a Leica confocal microscope under identical acquisition settings for all samples. For each heart, four non-overlapping fields were imaged. Imaging parameters (laser power, gain, and offset) were optimized using the sample with the highest fluorescence signal and subsequently kept constant across all images to allow quantitative comparison.

Quantitative analysis was performed using ImageJ (Fiji) with a custom macro. Thresholds for each fluorescence channel were adjusted consistently prior to segmentation. The following parameters were extracted for each image: signal area, fluorescence intensity, integrated density (area × intensity), percentage of signal-positive area, and number of nuclei. Fluorescence measurements were normalized to nuclear counts where indicated. Data were exported for statistical analysis.

## *Transmission Electron Microscopy*

Hearts were extracted from 8mo zebrafish (n = 5/genotype) and immediately immersed in McDowell fixative (6 mL/sample) for 4 hours at 4°C under constant agitation. Following fixation, wash buffer (0.1 M cacodylate buffer with 8% sucrose) was added to remove residual fixative.

Fixed samples were embedded in epoxy resin and semi-thin sections (500-1000 nm) were obtained using an ultramicrotome. Sections were stained with Toluidine Blue to facilitate tissue evaluation under light microscopy. The best-preserved regions of each sample were selected for subsequent processing for transmission electron microscopy (TEM).

For TEM analysis, ultra-thin sections (50-70 nm) were obtained from selected regions and placed on copper grids. Two grids were prepared per sample: one with Formvar coating and one without. Sections were contrasted with uranyl acetate and lead citrate before imaging. TEM analysis was performed at different magnifications ranging from 2000X to 50000X to evaluate sarcomere organization, intercalated disc integrity, mitochondrial distribution, and cellular infiltrates. Samples were observed with Orion software using Jeol 1011 transmission electron microscope equipped with a GATAN ORIUS™ camera for image acquire.

Acquired images were analysed to compare *wt* and *dspb^-/-^* mutant cardiac tissue, as differences in sarcomere alignment, Z-line integrity, mitochondrial morphology and the presence of interstitial structures. Gap junctions’ integrity, length and gap distance were assessed using QuPath (v0.5.1) software.

## *Gene expression analysis*

Both larvae and adult individuals were previously anesthetized dissolving 0.168 mg/mL tricaine buffered in Egg Water medium and then sacrificed through overdose by prolonged immersion (minimum 10 minutes) in tricaine (0.3 mg/mL dissolved in Egg Water medium). For adults, the heart was extracted, the tail was cut with the help of a scalpel and then a longitudinal cut was made at the ventral and dorsal level and the skin was extracted with the help of forceps. As for larvae, total RNA was extracted from larvae pools. Total RNA extraction was carried out using the mirVana^TM^ PARIS kit (Invitrogen) using the manufacturer's instructions and treated with DNase I using the RNase-Free DNase set kit (Qiagen). Next, the RNA concentration and purity were measured with an N-60 Nanophotometer (IMPLEN).

To synthesize first-strand cDNA, reverse transcription was performed using the iScript cDNA synthesis kit (BIO-RAD), using 1 μg of total RNA for larvae pools (pool of 20 larvae at 6 dpf, 10 larvae at 20 dpf) and skin, 0.5 μg RNA for tail and 0.3 μg RNA for heart tissue (pool of 3 hearts). The reaction included a priming step at 25 °C for 5 min, reverse transcription at 46 °C for 20 min, and reverse transcriptase inactivation at 95 °C for 1 min.

Quantitative real-time PCR (qPCR) was performed with the MyiQ® iCycler PCR Thermocycler and MyiQ iCycler Optical Module (Bio-Rad Laboratories®) using TB Green® Premix ExTaq™ II kit (Tli RNaseH Plus; Takara). Reaction mixtures were incubated for 10 s at 95°C, followed by 40 cycles of 5 s at 95°C and 20 s at 60°C, and finally 1 min at 95°C, 1 min 60°C, and 15 s at 95°C using the constitutive expression of the ribosomal protein S11 gene (*rps11*) as an endogenous control to normalize gene expression values. The primer sequences used are listed in Table S7. In all cases, each qPCR was performed with triplicate samples and repeated at least twice for each one of the genes to be studied. The relative quantification was carried out using the comparative CT method by evaluating the difference between the reference gene and the genes under study. A representative experiment out of three independent biological replicates is shown in figures.

| **Table S1.** Clinical severity in carriers of *DSP* truncating variants: impact of exercise intensity. |
| --- |
| \|  \| Sedentary \| Moderate/Intense \| p value \| \| --- \| --- \| --- \| --- \| \| n \| 32 (71.1%) \| 13 (28.9%) \|  \| \| Demographics \|  \|  \|  \| \| Male \| **8 (25.5%)** \| **10 (76.9%)** \| **0.002** \| \| Female \| 24 (75.0%) \| 3 (23.1%) \| \| Age of diagnosis \| **47.1 ± 13.7** \| **33.0 ± 13.5** \| **0.008** \| \| Follow-up (months) \| 108.2 ± 73.2 \| 87.8 ± 54.4 \| 0.346 \| \| Height (m) \| 1.6 ± 0.08 \| 1.7 ± 0.08 \| 0.072 \| \| Weight (Kg) \| 69.2 ± 12.5 \| 69.3 ± 12.0 \| 0.999 \| \| BSA (m2) \| 1.7 ± 0.1 \| 1.8 ± 0.1 \| 0.626 \| \| BMI (Kg/m2) \| 25.0 ± 3.9 \| 23.4 ± 3.4 \| 0.196 \| \| ESC sports classification \|  \|  \|  \| \| Skill&Power \|  \| 3 (23.0%) \|  \| \| Mixed&Endurance \|  \| 9 (69.2%) \|  \| \| DSPtv location*^1^ \|  \|  \|  \| \| Region 1 \| 5 (15.6%) \| 5 (23.0%) \|  \| \| Region 2 \| 24 (75.0 %) \| 8 (61.5%) \| 0.228 \| \| Region 3 \| 3 (9.4%) \| 0 (15.5%) \|  \| \| Reason for Diagnosis \|  \|  \|  \| \| Incidental \| 1 (3.1%) \| 1 (7.6%) \| 0.494 \| \| Symptoms \| **16 (50.0%)** \| **1 (7.6%)** \| **0.012** \| \| Family Screening \| 12 (37.5%) \| 8 (61.5%) \| 0.174 \| \| SD \| 1 (3.1%) \| 2 (15.3%) \| 0.191 \| \| FHSD \| 15 (46.8%) \| 6 (46.1%) \| 0.744 \| \| DCM \| 21 (65.6%) \| 8 (61.5%) \| 0.999 \| \| ND-LVC \| 11 (34.4%) \| 5 (38.5%) \| \| Symptoms \|  \|  \|  \| \| NYHA 1+2 \| 28 (87.5%) \| 13 (100.0%) \| 0.307 \| \| NYHA 3+4 \| 4 (12.5%) \| 0 (0.0%) \| \| Syncope \| 7 (21.8%) \| 0 (0.0%) \| 0.085 \| \| Chest pain \| 5 (15.6%) \| 0 (0.0%) \| 0.300 \| \| Palpitations \| 13 (40.6%) \| 0 (0.0%) \| **0.008** \| \| Tests \|  \|  \|  \| \| ECG - Abnormal \| 16 (50.0%) \| 9 (69.2%) \| 0.327 \| \| Holter** - NSVT \| 14 (58.3%) \| 5 (50.0%) \| 0.716 \| \| ECHO \|  \|  \|  \| \| LVEF (%) \| 45.5 ± 14.9 \| 52.1 ± 13.3 \| 0.181 \| \| LVEDd (mm) \| 54.4 ± 8.9 \| 55.5 ± 13.3 \| 0.813 \| \| MaxLVWT (mm) \| 11.0 ± 3.8 \| 9.9 ± 1.9 \| 0.198 \| \| LA (mm) \| 36.6 ± 10.0 \| 33.3 ± 5.6 \| 0.232 \| \| CMR*** \|  \|  \|  \| \| LVEF (%) \| 48.1 ± 13.2 \| 51.0 ± 11.6 \| 0.521 \| \| LVEDvol (ml) \| 178.7 ± 60.1 \| 200.0 ± 27.6 \| 0.183 \| \| RVEF (%) \| 50.0 ± 9.3 \| 55.6 ± 6.4 \| 0.059 \| \| RVEDvol (ml) \| 151.6 ± 47.2 \| 171.4 ± 32.2 \| 0.178 \| \| LGE \| 16 (76.1%) \| 10 (83.3%) \| 0.999 \| \| Devices \|  \|  \|  \| \| ICD \| 20 (62.5%) \| 8 (61.5%) \| 0.999 \| \| Events \|  \|  \|  \| \| No events \| 20 (62.5%) \| 10 (76.9%) \| 0.499 \| \| SD + RCA + ICD discharge \| 12 (37.5%) \| 3 (23.0%) \| 0.499 \| \| Transplant \| 2 (6.2%) \| 0 (0.0%) \| 0.999 \| |

BSA: Body Surface Area, BMI: Body Mass Index, DSPtv: DSP truncating variants, SD: sudden cardiac death, FHSD: Family history sudden death; DCM: Dilated Cardiomyopathy; ND-LVC: non-dilated left ventricular cardiomyopathy; NYHA: New York Heart Association Dyspnea Class, FHSD: family history of sudden death, NSVT: non-sustained ventricular tachycardia on Holter, LVEF: left ventricular ejection fraction, LVEDd: left ventricular end diastolic diameter, MaxLVWT: maximal left ventricular wall thickness, LA: left atrial diameter, CMR: cardiac magnetic resonance, LVEDvol: left ventricular end diastolic volume, RVEF: right ventricular ejection fraction, RVEDvol: right ventricular ejection fraction, LGE: late gadolinium enhancement, ICD: implantable cardioverter defibrillator, RCA: resuscitated cardiac arrest. *DSPtv location Region 1 = constitutive nonsense mediated decay competent region (c.1-3582), Region 2 = non-constitutive nonsense mediated decay competent region (c.3583-5379), Region 3 = constitutive nonsense mediated decay competent region (c.5380-8616), **Holter available in 77.7% of patients, ***CMR available in 73.3% of patients.

^1^E. T. Hoorntje et al., “Variant Location Is a Novel Risk Factor for Individuals With Arrhythmogenic Cardiomyopathy Due to a Desmoplakin (DSP) Truncating Variant,” Circ. Genomic Precis. Med., vol. 16, no. 1, p. E003672, Feb. 2023, doi: 10.1161/CIRCGEN.121.003672.

**Table S2.** Gene expression analysis in wt and *dspb*^-/-^ larvae and adult at different stages, to assess the expression levels of key genes in fundamental signalling pathways.

| Larvae | | | | | | | | | | | | |  |
| --- | --- | --- | --- | --- | --- | --- | --- | --- | --- | --- | --- | --- | --- |
|  | **6 dpf** | | | | **p value** | | **20 dpf** | | | | | **p value** | |
|  | **wt** | | ***dspb^-/-^*** | |  |  | **wt** | | ***dspb^-/-^*** | | |  |  |
| *ccnd1* | | 1.00 ± 0.03 | | 0.51 ± 0.17 | | <0.0001 | | 1.00 ± 0.14 | | 1.55 ± 0.25 | <0.0001 | | |
| *myc* | | 1.00 ± 0.05 | | 1.15 ± 0.11 | | ns | | 1.00 ± 0.15 | | 1.44 ± 0.56 | <0.0001 | | |
| *smad2* | | 1.00 ± 0.06 | | 0.48 ± 0.19 | | <0.0001 | | 1.00 ± 0.14 | | 1.20 ± 0.49 | ns | | |
| *smad3* | | 1.00 ± 0.20 | | 0.71 ± 0.53 | | ns | | 0.00 ± 0.37 | | 2.27 ± 1.45 | <0.0001 | | |
| *ccn2a* | | 1.00 ± 0.05 | | 0.14 ± 0.10 | | <0.001 | | 1.00 ± 0.08 | | 2.34 ± 1.23 | <0.0001 | | |
| *ccn2b* | | 1.00 ± 0.80 | | 0.30 ± 0.45 | | <0.0001 | | 1.00 ± 0.13 | | 0.71 ± 0.21 | <0.01 | | |
| Adults | | | | | | | | | | | | |  |
|  | **1 year-old** | | | | **p value** | | **2.5 year-old** | | | | | **p value** | |
|  | **wt** | | ***dspb^-/-^*** | |  |  | **wt** | | ***dspb*^-/-^** | | |  |  |
| *ccnd1* | | 1.00 ± 0.18 | | 0.12 ± 0.10 | | <0.0001 | | 1.00 ± 0.10 | | 0.06 ± 0.06 | <0.0001 | | |
| *myc* | | 1.00 ± 0.23 | | 0.23 ± 0.09 | | <0.0001 | | 1.00 ± 0.11 | | 0.29 ± 0.30 | <0.0001 | | |
| *smad2* | | 1.00 ± 0.22 | | 0.28 ± 0.11 | | <0.0001 | | 1.00 ± 0.41 | | 0.23 ± 0.22 | <0.0001 | | |
| *smad3* | | 1.00 ± 0.52 | | 0.32 ± 0.66 | | <0.001 | | 1.00 ± 0.47 | | 1.60 ± 3.44 | ns | | |
| *ccn2a* | | 1.00 ± 0.18 | | 0.08 ± 0.66 | | <0.0001 | | 1.00 ± 0.26 | | 0.41 ± 0.56 | <0.01 | | |
| *ccn2b* | | 1.00 ± 0.85 | | 0.07 ± 0.19 | | <0.0001 | | 1.00 ± 0.58 | | 0.6 ± 0.09 | ns | | |

*Number of experiments: 2 replicates for larvae stages; 3 replicates for 1* year-old*; 2 replicates for 2.5* year-old*; Sample size: pool of 20 larvae for 6 dpf, 10 larvae for 20 dpf, pool of 3 hearts for each adult group. (ns: not significant)*

**Table S3.** Cardiac function in wt and *dspb*^-/-^ adults, measured by echocardiography, showing the impact of moderate training.

|  | Pre-training | | Post-training | | WT pre *vs* dspb^‒/‒^ pre | | WT pre *vs* wt post | | dspb^‒/‒^ pre *vs* dspb^-/-^ post | |
| --- | --- | --- | --- | --- | --- | --- | --- | --- | --- | --- |
|  | wt (n=11)^1^ | dspb^-/-^ (n=11)^2^ | wt (n=10)^3^ | dspb-/-(n=10)^4^ | p value 1-2 | Hedges’ g 1-2 (95% CI) | p value 1-3 | Hedges’ g 1-3 (95% CI) | p value 2-4 | Hedges’ g 2-4 (95% CI) |
| Heart rate (beats/minute) | 131.00 ± 10.08 | 215.54 ± 11.32 | 111.00 ± 6.05 | 158.00 ± 11.81 | <0.001 | -2.29 (-3.36 – -1.21) | 1 | –0.69 (–1.55 – 0.16) | 0.003 | –2.30 (–3.20 – -1.40) |
| Respiratory frequency (breaths/minute) | 156.66 ± 14.39 | 218.40 ± 14.12 | 203.0 ± 25.30 | 240.90 ± 22.29 | 0.007 | -0.73 (-1.61 – 0.15) | 1 | 0.50 (–0.35 – 1.36) | 1 | 0.74 (0.05 – 1.43) |
| Systolic area (μm^2^) | 0.24 ± 0.01 | 0.18 ± 0.01 | 0.22 ± 0.01 | 0.21 ± 0.01 | 0.005 | 1.41 (0.48 – 2.35) | 1 | –0.42 (–1.25 – 0.41) | 0.450 | 0.79 (0.04 – 1.54) |
| Diastolic area (μm^2^) | 0.33 ± 0.02 | 0.23 ± 0.01 | 0.34 ± 0.02 | 0.29 ± 0.01 | <0.001 | 1.73 (0.75 – 2.71) | 1 | 0.11 (–0.72 – 0.93) | 0.004 | 1.14 (0.21 – 2.08) |
| Systolic volume (mm^3^) | 0.15 ± 0.01 | 0.10 ± 0.009 | 0.14 ± 0.01 | 0.13 ± 0.01 | 0.003 | 1.43 (0.49 – 2.36) | 0.69 | –0.29 (–1.11 – 0.54) | 0.038 | 0.99 (0.20 – 1.77) |
| Diastolic volume (mm^3^) | 0.25 ± 0.02 | 0. 14 ± 0.01 | 0.27 ± 0.02 | 0.20 ± 0.01 | <0.001 | 1.67 (0.70 – 2.64) | 1 | 0.230 (–0.59 – 1.05) | 0.003 | 1.273 (0.43 – 2.12) |
| Stroke Volume (mm^3^) | 0.10 ± 0.01 | 0.05 ± 0.003 | 0.13 ± 0.01 | 0.08 ± 0.005 | <0.001 | 2.06 (0.96– 3.16) | 0.202 | 0.68 (–0.17 – 1.53) | <0.001 | 1.32 (0.37 – 2.26) |
| Cardiac Output/HR (ml/min) | 0.10 ± 0.01 | 0.05 ± 0.003 | 0.13 ± 0.02 | 0.08 ± 0.005 | <0.001 | 2.06 (0.96– 3.16) | 0.202 | 0.46 (–0.37 – 1.29) | <0.001 | 0.42 (–0.42 – 1.26) |
| Fractional Area of Change/HR (%) | 0.23 ± 0.03 | 0.11 ± 0.01 | 0.32 ± 0.03 | 0.18 ± 0.01 | 0.003 | 1.63 (0.61 – 2.65) | 0.031 | 1.26 (0.34 – 2.17) | <0.001 | 0.76 (–0.11 – 1.62) |
| Ejection fraction (%) | 38.75 ± 1.65 | 31.33 ± 2.07 | 46.48 ± 2.34 | 37.27 ± 1.61 | 0.012 | 1.2 (0.24 – 2.16) | 0.022 | 1.25 (0.34 – 2.16) | 0.038 | 0.82 (0.14 – 1.50) |
| Apex to base distance (mm) (systolic) | 0.28 ± 0.01 | 0.26 ± 0.01 | 0.30 ± 0.01 | 0.29 ± 0.009 | 1 | 0.40 (–0.44 – 1.25) | 1 | 0.39 (–0.43 – 1.23) | 0.522 | 0.47 (–0.13 – 1.08) |
| Apex to base distance (mm) (diastolic) | 0.35 ± 0.01 | 0.32 ± 0.01 | 0.38 ± 0.01 | 0.35 ± 0.01 | 0.894 | 0.59 (–0.27 – 1.45) | 1 | 0.49 (–0.341 – 1.33) | 0.826 | 0.63 (–0.05 – 1.31) |
| Ventricular entry diameter (mm) (systolic) | 0.25 ± 0.008 | 0.21 ± 0.01 | 0.24 ± 0.01 | 0.22 ± 0.01 | 0.604 | 0.98 (0.09 – 1.88) | 1 | –0.12 (–0.99 – 0.76) | 1 | 0.48 (–0.12 – 1.09) |
| Ventricular entry diameter (mm) (diastolic) | 0.32 ± 0.01 | 0.25 ± 0.01 | 0.30 ± 0.01 | 0.28 ± 0.01 | 0.003 | 1.68 (0.68 – 2.68) | 1 | –0.11 (–0.96 – 0.78) | 0.909 | 0.56 (–0.06 – 1.18) |
| Doppler A wave (mm/s) | 66.37 ± 5.96 | 38.74 ± 4.26 | 69.78 ± 6.71 | 40.50 ± 4.64 | 0.004 | 1.55 (0.55 – 2.55) | 1 | 0.02 (–0.98 – 1.02) | 1 | 0.08 (–0.87 – 1.04) |
| Doppler VAo wave (mm/s) | -18.40 ± 2.58 | -11.47 ± 1.81 | -24.59 ± 3.10 | -20.35 ± 2.04 | 0.831 | –1.16 (–2.49 – 0.16) | 0.722 | –0.39 (–1.58 – 0.79) | 0.008 | –1.11 (–1.97 – -0.24) |
| Doppler E wave (mm/s) | 14.12 ± 2.06 | 12.45 ± 2.10 | 9.88 ± 1.14 | 8.74 ± 1.07 | 1 | 0.25 (–0.70 – 1.20) | 0.955 | –0.59 (–1.60 – 0.42) | 1 | –0.97 (–1.92 – -0.01) |
| VE/VA (ratio/BodyLenght) | 0.07 ± 0.007 | 0.10 ± 0.01 | 0.06 ± 0.01 | 0.09 ± 0.009 | 0.366 | –0.84 (–1.84 – 0.15) | 1 | –0.38 (–1.47 – 0.71) | 1 | –0.16 (–1.09 – 0.78) |

Values are presented as mean ± SEM. Statistical analyses were performed using one-way ANOVA for normally distributed variables with Bonferroni post-hoc correction, or Mann–Whitney U tests for non-parametric variables, as indicated. The table reports global *p*-values (ANOVA or Kruskal–Wallis) and post-hoc pairwise comparisons. Effect sizes are reported as Hedges’ g with 95% confidence intervals (CI). Hedges’ g was calculated as the bias-corrected standardized mean difference between groups, using pooled standard deviations and adjusting for small-to-moderate sample sizes. Effect sizes were interpreted according to conventional thresholds (small ≈ 0.2, moderate ≈ 0.5, large ≥ 0.8). Positive values indicate higher measurements in *dspb*^–/–^ compared with wt.HR: Heart rate

**Table S4.** Haematoxylin-Eosin staining and Macroscopical study of zebrafish heart at different ages - comparison between wt and *dspb*^-/-^

|  | Young | | | Adult | | | Old | | |
| --- | --- | --- | --- | --- | --- | --- | --- | --- | --- |
|  | wt | *dspb^-/-^* | p value | wt | *dspb^-/-^* | p value | wt | *dspb^-/-^* | p value |
| Length (mm/BMI) | 13.26 ± 3.56 | 9.19 ± 2.58 | 0.02 | 10.58 ± 1.98 | 9.58 ± 0.78 | ns | 5.00 ± 1.06 | 7.77 ± 4.13 | ns |
| Width (mm/BMI) | 13.00 ± 2.41 | 8.16 ± 1.45 | 0.015 | 9.46 ± 2.35 | 8.06 ± 0.48 | ns | 4.7 ± 1.49 | 7.10 ± 3.38 | ns |
| Perimeter (mm/BMI) | 43.67 ± 9.94 | 28.90 ± 5.59 | 0.008 | 34.57 ± 8.27 | 30.04 ± 1.53 | ns | 17.21 ± 3.96 | 25.92 ± 13.63 | ns |
| Total Area (mm^2^/BMI) | 28.74 ± 12.54 | 17 ± 5.25 | 0.02 | 19.94 ± 7.28 | 16.31 ± 1.36 | ns | 6.88 ± 2.31 | 14.92 ± 9.86 | ns |
| Compact Tissue % | 82.99 ± 4.24 | 82.36 ± 4.54 | ns | 84.14± 5.18 | 82.90 ± 11.93 | ns | 85.17 ± 7.16 | 83.21 ± 9.38 | ns |
| Fatty tissue % | No adipocytes were observed | | - | No adipocytes were observed | | - | 2.99 ± 1.74 | 3.44 ± 1.37 | ns |

n=5 individuals for each genotype, for each age group: young (10 months old), adult (1.5 years old) and old (2.5 years old)

|  | Untrained | | Trained | p value  1-2 | p value  2-3 |
| --- | --- | --- | --- | --- | --- |
|  | **wt (n=4)^1^** | ***dspb^-/-^* (n=4)^2^** | ***dspb^-/-^*(n=4)^3^** |  |  |
| Total distance moved (m) | 507.25 ± 124.18 | 98.28 ± 55.7 | 160.89 ± 22.54 | 0.002 | 0.90 |
| Mobile - Cumulative Duration (s) | 629.20 ± 83.16 | 164.67 ± 155.33 | 138.87 ± 35.82 | 0.02 | 0.90 |
| Immobile - Frequency | 2239 ± 665.10 | 5400 ± 788.80 | 1310 ± 289.40 | 0.02 | 0.00108 |
| Immobile - Cumulative Duration (s) | 287.51 ± 84.4 | 849.39 ± 14.69 | 179.84 ± 14.98 | 0.007 | 0.000001 |

**Table S5.** Endurance assays for previously trained and untrained fish, parameters collected by EthoVision XT software.

**Table S6. Summary of the main dsp-deficient zebrafish models and the experimental approaches used to date.** Each row reports the developmental stage at which analyses were conducted, along with the corresponding phenotypes or molecular findings. Results are categorized by the type of alteration observed compared to wildtype siblings.

|  | *dspa/dspb* morphants  (Giuliodori et al., 2018) | *dspa* ENU mutants and *dspb* crispants  (Celeghin et al., 2023) | | *dspb* crispant (our model) |
| --- | --- | --- | --- | --- |
| Phenotype and Morphology | - 2 dpf: mild developmental delay (brightfield microscopy) | | - 3 dpf: cardiac dilation, pericardial effusion, developmental delay, increased L-plastin+ cells (morphology, immunofluorescence, confocal microscopy) | - 3 dpf: smaller larvae, abnormal yolk sac, delayed hatching (morphometrics) - 10 mpf: significantly smaller hearts (macroscopic measurement) |
| Cardiac Function | - 2 dpf: bradycardia *(high-speed video microscopy)* | | - 2-7 dpf: bradycardia - 3 dpf: reduced ventricular contractility and ejection fraction *(high-speed video microscopy)* | - 3-6 dpf: tachycardia *(video microscopy)* - 8 mpf: tachycardia, tachypnea, ↓ A-wave, ↓ stroke volume, smaller ventricle size *(echocardiography)* - 8-9 mpf: partial functional recovery post-exercise *(long-term training + echocardiography)* |
| Molecular Analysis | - 2 dpf: altered Wnt, TGFβ, Hippo signaling *(qRT-PCR)* | | - 3 dpf: reduced dspa/dspb mRNA and proteins *(qRT-PCR, Western blot)* - 3 dpf: signaling dysregulation *(qRT-PCR: ccnd1, myc, smad2/3, ccn2a/b)* - 1 ypf: regenerative/fibrotic expression profile *(qRT-PCR)* | - 6 and 20 dpf: signaling dysregulation *(qRT-PCR: ccnd1, myc, smad2/3, ccn2a/b)* - 20 dpf: ↓ dspb, ↓ pkp2 *(qRT-PCR)* - 1 ypf: ↑ dspa, ↓ dspb, ↑ pkp2 in heart/skin/tail *(qRT-PCR)* - 1 and 2.5 ypf: global ↓ expression of six target genes in cardiac tissue *(qRT-PCR)* |
| Cellular and Cardiac Structure | - 2 dpf: pale desmosomes, detached cells *(TEM)* | | - 3 and 6 mpf: pale desmosomes, ↑ extracellular space *(TEM)* - 6 mpf: myocardial thinning, trabecular disorganization *(histology, resting)* - 9 mpf: myocardial thickening, no fibrosis *(histology)* - 3-6 mpf: worsened structure under training *(histology)* | - 8 mpf: more nuclei (inflammatory), wider desmosomal gaps, sarcomeric disarray *(semi-/ultrathin TEM)* - 2.5 ypf: adipocytes present in heart tissue, no fibrosis or compaction defects *(histology)* |
| Survival and Training Resonse | - Not evaluated | | - 0-30 dpf: lethality in homozygous mutants *(survival tracking)* - 5 dpf: reduced swimming performance *(macroscopic observation)* - 3-13 dpf: increased mortality under mild training *(training protocol)* | - 0-30 dpf: reduced survival *(survival tracking)* - 9 mpf: low endurance in untrained fish, partial recovery with prior training *(endurance protocol)* |
| Therapeutic Intervention | - Not evaluated | | - 1-3 dpf: partial rescue of survival, development, and bradycardia *(SB216763 treatment)* | - Not evaluated |

**Table S7.** Primer list for genotyping PCR reactions, qPCR and CRISPR/Cas9 DNA oligomers designed for genomic editing

| Gene/reference sequence | Forward  Primer sequence (5´—> 3´) | Reverse  Primer sequence (5´—> 3´) |
| --- | --- | --- |
| Genotyping primers | | |
| *dspb* | GGTGGTTCGGCAGTATGAAT | GTAGTAATCGCTTGAGCGGG |
| qPCR primers | | |
| *dspb/NM_001423427.1* | CATCGGCCGCATCAACTCAC | GTCGAAGTGCTGCTGTGCTC |
| *rsp11/NM_213377.1* | TCAGAACGAGAGGGCTTAT | CATCAATAGCTTCTCTGGG |
| *dspa/NM_001423755.1* | CTGCTGCCGAAGCCATGAAG | CCTTTGCCATCAAGCCACCC |
| *pkp2/NM_001113433.1* | CAGGTGTTCAGTCTCGGCCA | GCGCATGAAGTCCCTCTCCT |
| *ccnd1/NM_131025.4* | CCAACTTCCTCTCGCAAGTC | TGGTCTCTGTGGAGATGTGC |
| *myc/NM_131412.1* | AGAAAGCTGGAGTCCTCGAC | CTGCTGCAGTGTGTTCAGC |
| *smad2/NM_001290015.1* | TCATGTCATCTACTGCCGCC | GTCTTGGCACGAGAACAGGA |
| *smad3/NM_131571.2* | CTATCAGCGGGTCGAGACAC | AGTTGCTCTGGGGTTCGATG |
| *ccn2a/NM_001015041.2* | CTCCCCAAGTAACCGTCGTA | CTACAGCACCGTCCAGACAC |
| *ccn2b/NM_001102573.1* | CCCACAAGAAGACACCTTCC | ATTCGCTCCATTCAGTGGTC |
| CRISPR/Cas9 DNA oligomers for gRNA | | |
| *dspb*-specific oligo | TAATACGACTCACTATA**GGCCACTTACAGCGCTGGAC**GTTTTAGAGCTAGAAATAGCAAG | |
| constant oligo | AAAAGCACCGACTCGGTGCCACTTTTTCAAGTTGATAACGGACTAGCCTTATTTTAACTTGCTATTTCTAGCTCTAAAAC | |


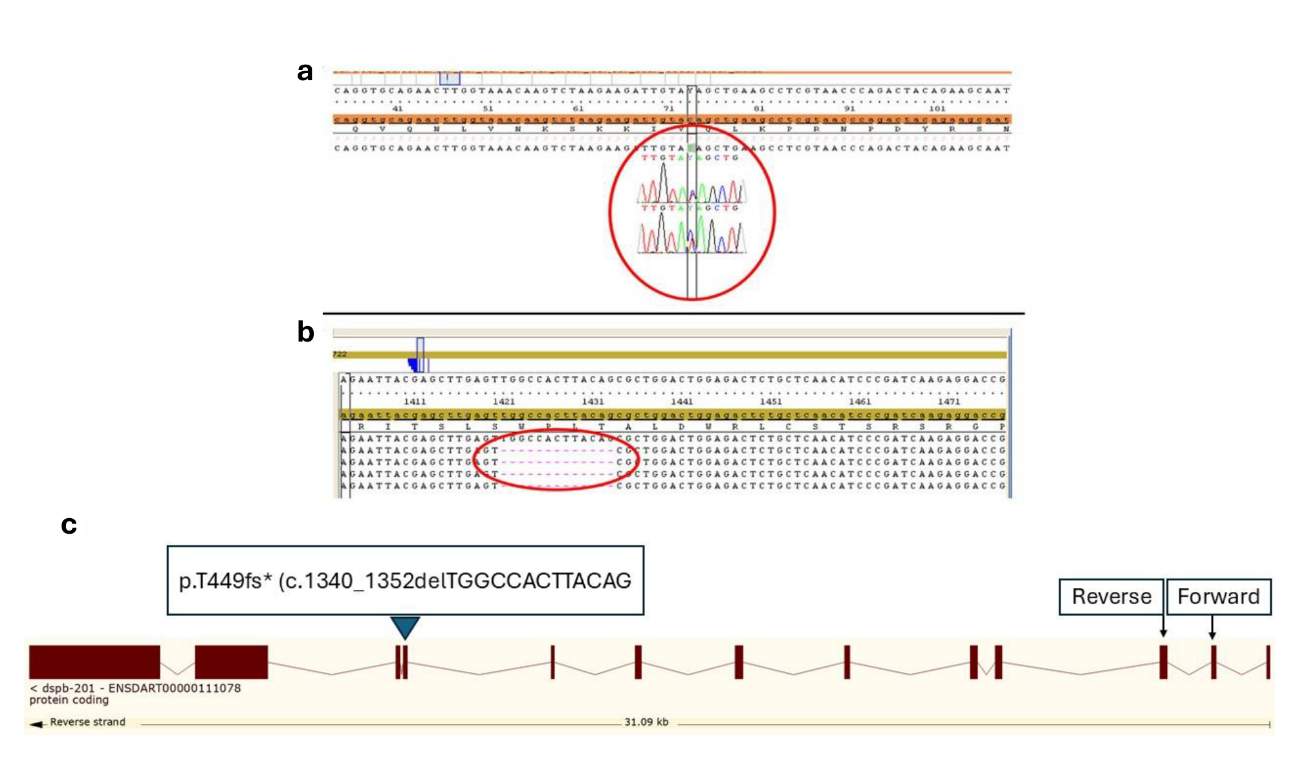


**Fig. S1**. Genotyping of human (**a**) vs. zebrafish (**b**) *dspb*^-/-^ mutants and wt. (**c**) Diagram of the zebrafish *dspb* genomic structure showing the exon organization. Forward and reverse arrows indicate the primer pairs used for qPCR amplification. The triangle marks exon 10, where the identified variant is located.


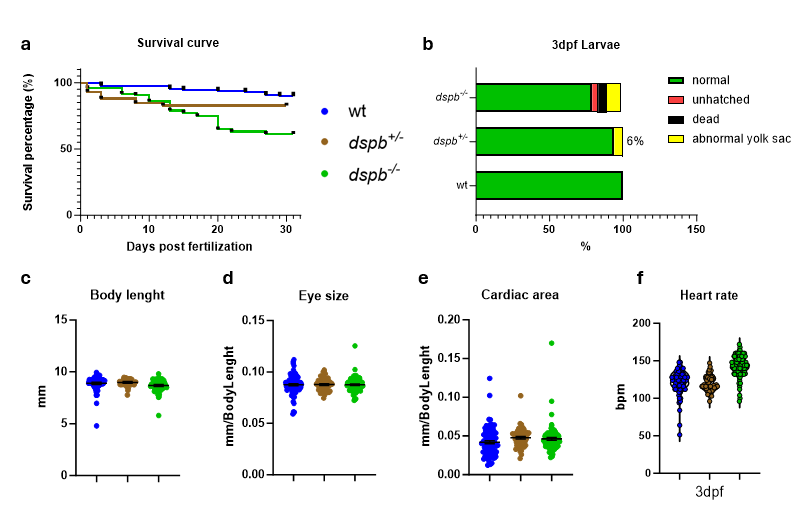


**Fig. S2. Phenotypic characterization of heterozygous *dspb^+/^*^−^ zebrafish.** (**a**) Survival analysis of *dspb^+/−^* and wt groups showed no significant differences in survival rates (wt 89.76% vs. *dspb^+/−^* 82.75%) (HR 1.9; 95% CI: 0.8–4.9; p>0.05). Test: Kaplan–Meier survival analysis with Log-rank (Mantel–Cox) and Hazard Ratio (Mantel–Haenszel). (**b**) While 100% of wt embryos displayed normal morphology, the *dspb^+/−^* group consisted of 94% normal larvae and 6% showing yolk sac abnormalities. (**c**) Body length measurements showed no significant differences between wt and *dspb^+/−^* larvae (wt 8.89 ± 0.06 mm vs. *dspb^+/−^* 8.97 ± 0.04 mm; p>0.05). (**d**) Eye size (wt 0.08 ± 0.0009 mm vs. *dspb^+/−^* 0.08 ± 0.0008 mm; p>0.05) and (**e**) cardiac area (wt 0.04 ± 0.001 mm² vs. *dspb^+/−^* 0.04 ± 0.001 mm²; p>0.05), both normalized to body length, showed no statistically significant differences. (**f**) Heart rate at 3 dpf showed comparable values between groups (wt 122.10 ± 1.53 bpm vs. *dspb^+/−^* 120.10 ± 1.40 bpm; p>0.05). Sample sizes: wt, n=100; *dspb^+/-^*n=54. Test: Two-way ANOVA with Bonferroni post hoc.


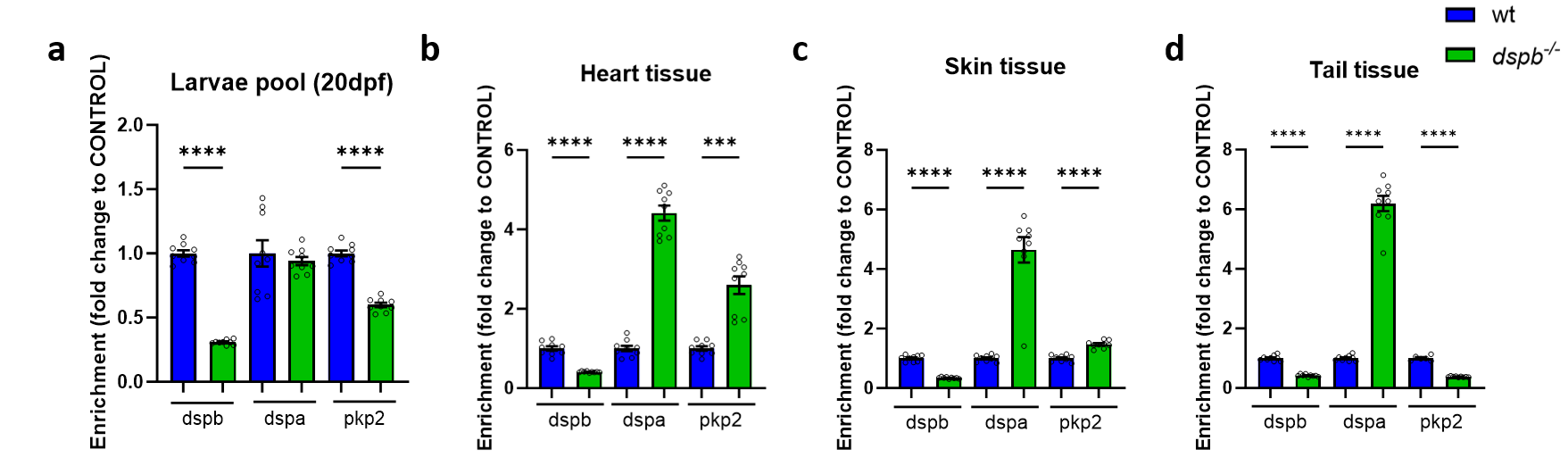


**Fig. S3.** Gene expression analysis in wt and *dspb^-/-^*mutants using (**a**) larvae pool at 20 days post fertilization and (**b,c,d**) adults in different types of tissue. Genes analysed were desmoplakin B, desmoplakin A and plakophilin 2. To determine the enrichment of each gene, constitutive gene rps11 was used as control to calculate the fold change (**** p<0.0001) Error bars: SEM. Test: Student’s t-test. Number of experiments: 3 replicates.

**
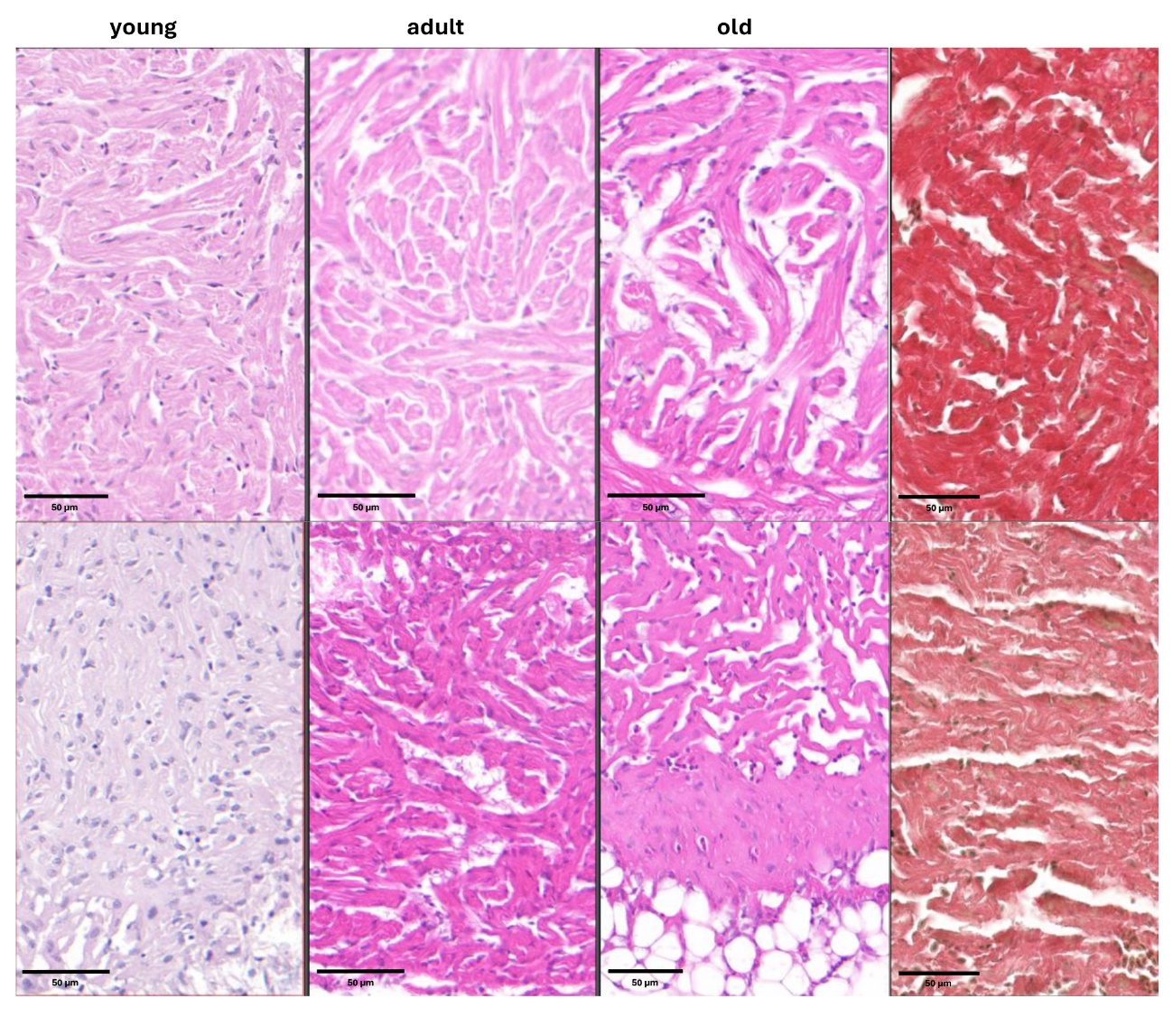
**

**Fig. S4**. Hematoxylin-Eosin staining (panels 1-3, top and bottom) analysis and comparison between wt and *dspb^-/-^* at three stages: young, adult and old age (**top panels** – wt, **bottom panels** – *dspb^-/-^*). Presence of adipocytes can be observed at old age. Masson trichrome staining images (last panels, top and bottom) and comparison between wt and *dspb^-/-^* at young age show no signs of fibrosis.

**
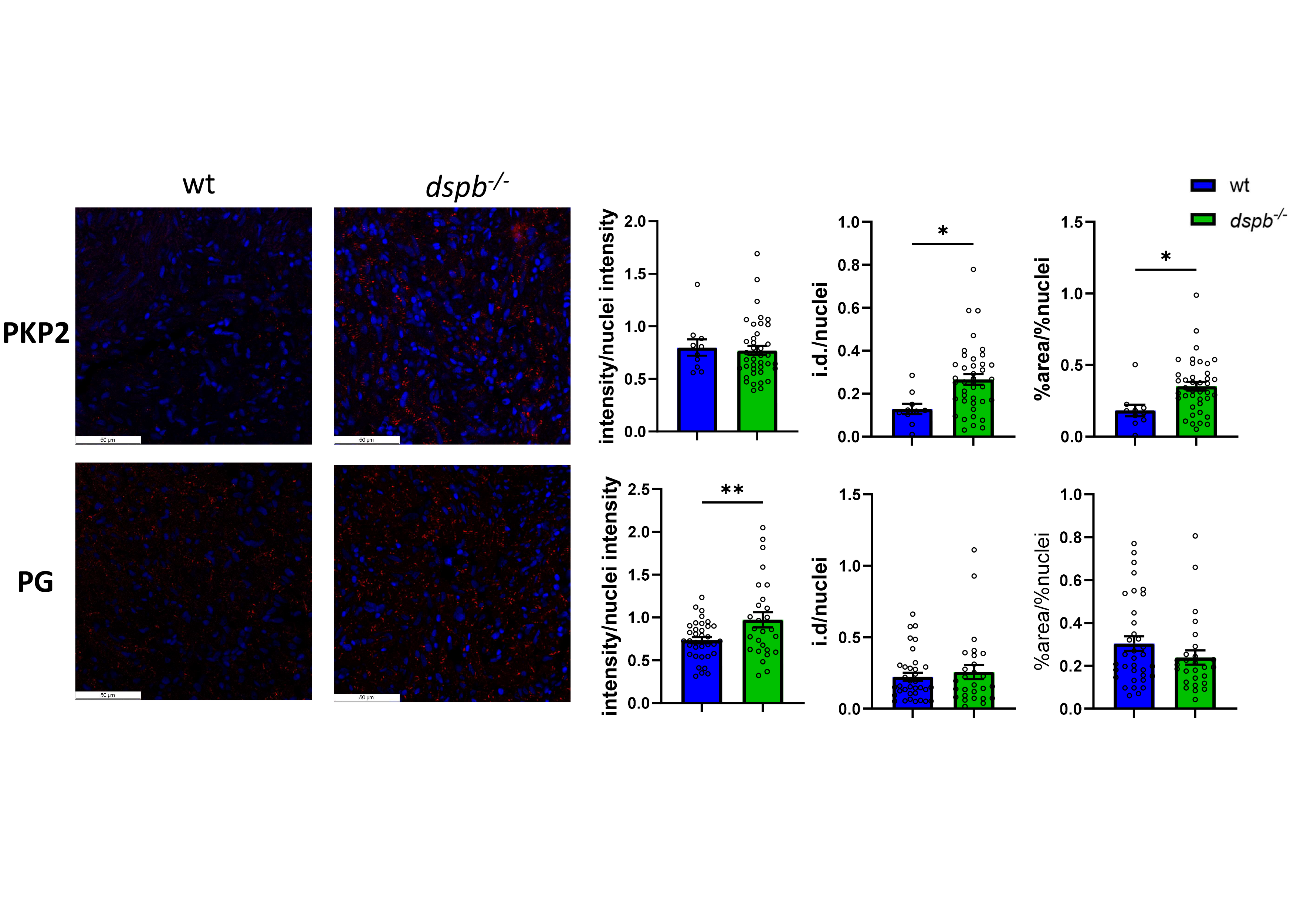
 Fig. S5. *Immunofluorescence analysis of PKP2 and PG in wt and dspb^-/-^ zebrafish hearts.*** Representative immunofluorescence images of adult zebrafish myocardium stained for PKP2 or PG (red) and nuclei (Hoechst, blue) in wt and *dspb^–/–^* hearts. Quantitative analysis (right panels) shows that PKP2 integrated density and PKP2-positive area normalized to nuclear area are significantly increased in *dspb^–/–^* hearts compared with WT, whereas the PKP2 intensity-to-nuclear-intensity ratio does not differ between genotypes. In contrast, PG displays a significantly higher intensity-to-nuclear-intensity ratio in *dspb^–/–^* hearts, while integrated density and protein-positive area normalized to nuclear area are not significantly different. Data are presented as mean ± SEM. Sample size: n = 11 WT and n = 9 *dspb^–/–^* hearts, with four imaging fields analyzed per sample. Statistical analysis was performed using unpaired Student’s t-test (*p < 0.05, **p < 0.01). PKP2, plakophilin-2; PG, plakoglobin; i.d., integrated density.
